# Hypertonic Saline Suppresses NADPH Oxidase-Dependent Neutrophil Extracellular Trap Formation and Promotes Apoptosis

**DOI:** 10.3389/fimmu.2018.00359

**Published:** 2018-03-08

**Authors:** Ajantha Nadesalingam, Jacky H. K. Chen, Armin Farahvash, Meraj A. Khan

**Affiliations:** ^1^Program in Translational Medicine, Peter Gilgan Centre for Research and Learning, The Hospital for Sick Children, Toronto, ON, Canada; ^2^Department of Laboratory Medicine and Pathobiology, University of Toronto, Toronto, ON, Canada

**Keywords:** hypertonic saline, NaCl, neutrophil extracellular traps, NOX2-dependent NETosis, lipopolysaccharide-induced NETosis, Gram-negative and -positive bacteria-induced NETosis, cystic fibrosis

## Abstract

Tonicity of saline (NaCl) is important in regulating cellular functions and homeostasis. Hypertonic saline is administered to treat many inflammatory diseases, including cystic fibrosis. Excess neutrophil extracellular trap (NET) formation, or NETosis, is associated with many pathological conditions including chronic inflammation. Despite the known therapeutic benefits of hypertonic saline, its underlying mechanisms are not clearly understood. Therefore, we aimed to elucidate the effects of hypertonic saline in modulating NETosis. For this purpose, we purified human neutrophils and induced NETosis using agonists such as diacylglycerol mimetic phorbol myristate acetate (PMA), Gram-negative bacterial cell wall component lipopolysaccharide (LPS), calcium ionophores (A23187 and ionomycin from *Streptomyces conglobatus*), and bacteria (*Pseudomonas aeruginosa* and *Staphylococcus aureus*). We then analyzed neutrophils and NETs using Sytox green assay, immunostaining of NET components and apoptosis markers, confocal microscopy, and pH sensing reagents. This study found that hypertonic NaCl suppresses nicotinamide adenine dinucleotide phosphate oxidase (NADPH2 or NOX2)-dependent NETosis induced by agonists PMA, *Escherichia coli* LPS (0111:B4 and O128:B12), and *P. aeruginosa*. Hypertonic saline also suppresses LPS- and PMA- induced reactive oxygen species production. It was determined that supplementing H_2_O_2_ reverses the suppressive effect of hypertonic saline on NOX2-dependent NETosis. Many of the aforementioned suppressive effects were observed in the presence of equimolar concentrations of choline chloride and osmolytes (d-mannitol and d-sorbitol). This suggests that the mechanism by which hypertonic saline suppresses NOX2-dependent NETosis is via neutrophil dehydration. Hypertonic NaCl does not significantly alter the intracellular pH of neutrophils. We found that hypertonic NaCl induces apoptosis while suppressing NOX2-dependent NETosis. In contrast, hypertonic solutions do not suppress NOX2-independent NETosis. Although hypertonic saline partially suppresses ionomycin-induced NETosis, it enhances A23187-induced NETosis, and it does not alter *S. aureus*-induced NETosis. Overall, this study determined that hypertonic saline suppresses NOX2-dependent NETosis induced by several agonists; in contrast, it has variable effects on neutrophil death induced by NOX2-independent NETosis agonists. These findings are important in understanding the regulation of NETosis and apoptosis in neutrophils.

## Introduction

Hypertonic saline has shown to be an effective therapy for a number of illnesses including cystic fibrosis (CF) and sepsis (1–3). CF patients suffer from dry, clogged, and chronically infected lungs (4). In CF patients, hypertonic saline is routinely administered to promote mucociliary clearance, surface liquid hydration, and the modulation of immune functions (5–11). Studies have shown that neutrophil infiltration occurs in the airways of CF patients, contributing to disease exacerbation (12). However, the mechanism by which hypertonic NaCl promotes mucociliary clearance is not clearly understood. In particular, the effects of hypertonic NaCl on reactive oxygen species (ROS) production and neutrophil death have not been extensively researched.

Neutrophils, the most abundant innate immune cells, play a vital role in physiological and pathological conditions such as inflammatory infections (13–15). In recent years, the discovery of NETosis, an alternate form of programmed cell death, has determined the role of inflammation in neutrophil-facilitated bacterial clearance (16). NETosis is the formation of neutrophil extracellular traps (NETs), which are decondensed DNA structures adorned with numerous antimicrobial peptides (17, 18). So far, a nicotinamide adenine dinucleotide phosphate (NADPH) oxidase (NOX2)-dependent NETosis and a NOX2-independent NETosis pathway have been described (19–21). The formation of NETs is beneficial in eradicating infections. However, NET production is a double-edged sword; the dysregulation or excess NET release can cause local tissue injuries during inflammatory diseases (22, 23). The regulatory mechanisms of NETosis are still not completely understood.

We, and others, have shown that phorbol myristate acetate (PMA), various bacteria (e.g., *Pseudomonas aeruginosa, Staphylococcus aureus*), and bacterial components [e.g., lipopolysaccharides (LPSs)] induce NOX2-dependent NETosis (20, 21, 24). A key step in this type of NETosis is the production of ROS by the activation of the NOX enzyme (25). Furthermore, the activation of MAP kinases and neutrophil elastase cleavage leads to chromatin decondensation and the release of NETs. In contrast, the more rapid NOX2-indpendent pathway of NETosis is driven by calcium influx and other factors (19). This pathway of NETosis is induced by calcium ionophores, A23187 and ionomycin. Ionomycin is secreted by the Gram-positive bacteria, *Streptomyces conglobatus*. Some studies have shown that the Gram-positive bacteria *Staphylococcus aureus* induces NOX2-independent NETosis (26, 27). Patients with CF have an elevated presence of thick mucus, DNA, proteins, and bacteria that obstruct their airflow (28–30). More recently, some studies have shown that the extracellular DNA levels correlate with neutrophil counts, which is used to assess inflammation and lung disease severity (28, 31, 32).

This study focuses on elucidating the regulatory effects of hypertonic saline (NaCl) on NETosis induced by various NOX2-dependent and -independent agonists. The results indicate that hypertonic NaCl suppresses NADPH oxidase-mediated ROS production and subsequently suppresses NET release. Next, we determine which element—sodium or chloride—is responsible for these suppressive effects. Thus, we replaced sodium chloride with equimolar concentrations of choline chloride. Results show similar effects of suppression on NETosis. Subsequently, this suppression in NETosis is reversed by administering hydrogen peroxide, two hours after salt treatment. Furthermore, the increasing concentrations of non-ionic osmolytes (d-mannitol and d-sorbitol) also suppress ROS production and PMA-, LPS-, and ionomycin-mediated NETosis. Immunostaining of the apoptotic marker, cleaved Caspase 3 (cCasp-3), shows that hypertonic saline suppresses NETosis and promotes apoptosis as an alternate pathway of programmed cell death. This finding suggests that the tonicity and osmolarity of NaCl are key players in regulating NETosis by inducing apoptosis.

Collectively, these data show that hypertonic NaCl has a suppressive effect on NOX2-dependent and ionomycin-induced NOX2-independent pathways of NET formation. This suppression of NETotic cell death is coupled with a promotion of the apoptotic pathway of cell death. This information could facilitate the understanding and planning of novel salt treatments in neutrophil associated inflammatory diseases.

## Materials and Methods

### Ethical Approval

The Research Ethics Board (REB) of the Hospital for Sick Children has approved present study protocols. All the study participants (healthy donors) signed the informed consent. All related methods were performed in accordance with the REB guidelines and regulations.

### Chemicals and Media Preparation

Chemicals and reagents, including PMA, LPS (0111:B4 and O128:B12), A23187, ionomycin, NaCl, d-mannitol, d-sorbitol, and choline chloride were obtained from Sigma (St. Louis, MO, USA), unless otherwise stated. RPMI medium (Wisent Inc.) with 10 mM HEPES (pH 7.2) has been used as the standard medium for all experiments. Without modification, this medium contains 103.44 mM NaCl and 109 mM total chloride. In the clinical treatment of CF, the nebulization of up to 7% hypertonic saline results in significant mucociliary clearance (33, 34). In most of the clinical settings, approximately 3% hypertonic saline has been used in the pulmonary treatment of CF (35). In our study, 3% hypertonic saline corresponds to ~513 mM NaCl concentration. To test whether this range of NaCl influences NETosis, eight different concentrations (109, 115, 121, 134, 159, 209, 309, and 509 mM) of NaCl, or choline chloride were prepared in RPMI medium. The media with altered concentrations were used for NETosis, ROS, and different assays. To test the effects of tonicity and osmolarity on neutrophils, NETosis kinetics and ROS generation were assessed in the presence of non-ionic osmolytes d-mannitol and d-sorbitol. d-mannitol and d-sorbitol are known to increase osmolarity.

### Human Primary Neutrophil Isolation

Human peripheral blood was drawn from healthy donors and collected in K2 EDTA blood collection tubes (Becton, Dickinson, Co.). Neutrophils were isolated from whole blood using PolymorphPrep (Axis-Shield) according to the manufacturer’s instructions with minor modifications as reported in previous studies (16, 21, 23, 25). After washing the neutrophils, residual red blood cells were lysed using a 0.2% (w/v) NaCl hypotonic solution for 30 s. Subsequently, an equal volume of 1.6% (w/v) NaCl solution with 20 mM HEPES buffer was added. In order to eliminate red blood cell debris, cells were washed twice with 0.85% (w/v) NaCl containing 10 mM HEPES. Then, neutrophils were resuspended in RPMI medium (Invitrogen) with 10 mM HEPES (pH 7.2) for further use. Finally, purity of the neutrophils was ascertained; cell density was quantified using a hemocytometer, cell purity by Cytospin preparations, and cell viability determined by trypan blue dye. Neutrophil preparations having >95% purity were used in all the experiments reported in this study. The normal neutrophil count was defined as 1−2 million purified neutrophils per mL of the collected blood. Donors who had high neutrophil count were excluded to avoid potential baseline activation due to any underlying inflammatory conditions.

### NETosis Kinetics by Sytox Green Assay

NETosis (% DNA release) kinetics were assessed using Sytox Green, a cell impermeable DNA binding dye. The Sytox Green dye exhibits >500-fold fluorescence enhancement upon binding with nucleic acids. Therefore, a measure of the fluorescence emitted by the dye has been used as a proxy for DNA release. 50,000 cells in a volume of 50 µl cell suspension with 5 µM Sytox Green dye were seeded into 96-well plates. The salt and different non-ionic osmolytes (d-mannitol, d-sorbitol) conditions of corresponding wells were altered by adding an equal volume of RPMI media with different salt and osmolyte concentrations (109, 115, 121, 134, 159, 209, 309, and 509 mM). Immediately after changing the salt or osmolytes concentrations of the media, cells were either left untreated (−ve control) or activated by NOX2-dependent agonists (25 nM PMA; 25 µg/mL 0111:B4 LPS and 5 μg/mL O128:B12), NOX2-independent agonists (4 µM A23187; 5 µM ionomycin), or bacteria [*Pseudomonas aeruginosa* at 20 multiplicity of infection (MOI), *Staphylococcus aureus* at 20 MOI]. After cell activation, the changes in the fluorescence intensities were measured every 30 min for up to 210 min using a POLARstar OMEGA (BMG Labtech) at 504 nm excitation and 523 nm emission. To calculate the amount of NETosis as a function of percent DNA release, the fluorescence reading at time zero was subtracted from the fluorescence reading at every time point. This value was then divided by the amount of total DNA, as determined by the fluorescence values of neutrophils lysed with 0.5% (w/v) Triton X-100. These experiments were repeated replacing NaCl with, d-mannitol, d-sorbitol, or choline chloride. Assays were excluded if baseline activation was >30%.

### Immunofluorescence Confocal Imaging

Immunofluorescence confocal imaging was performed to confirm the induction of NETosis. 50,000 neutrophils in a volume of 100 µl were seeded into eight-well chamber slides (BD Falcon). Salt or osmolyte (d-mannitol or d-sorbitol) concentrations of the neutrophils were changed by adding equal volume of RPMI with different concentrations of salt and d-mannitol to adjust for 109, 115, 121, 134, 159, 209, 309, and 509 mM into corresponding wells. Neutrophils in different salt media were either left untreated (−ve control) or activated by PMA or LPS, and incubated at 37°C for 120 min. After the incubation time, cells were fixed with 4% (w/v) PFA overnight at 4°C. Following three washes with phosphate-buffered saline (PBS), cells were permeabilized using 0.05% (w/v) Triton X-100 for 15 min. Following another three washes with PBS, cells were blocked for 1 h using 5% (w/v) bovine serum albumin. Furthermore, the cells were washed thrice using PBS. Then, cells were incubated with a primary antibody against myeloperoxidase (MPO, ab25989, Abcam) at a 1:1,000 concentration for 1.5 h. The isotype controls, mouse IgG (eBioscience 14-4714-81; 1:100 dilution) was used to confirm the MPO signals and mouse MPO antibody specificity. After three washes, a secondary antibody and nucleic acid-binding DAPI dye (Invitrogen; at 1:100 dilution) was added for 45 min. The secondary antibody was a donkey anti-mouse (488 nm, ab98766, Abcam) at a concentration of 1:1,000. Cells were then washed three more times, and then mounted for imaging. Confocal images were taken with an Olympus IX81 inverted fluorescence microscope with a Hamamatsu C9100-13 back-thinned EM-CCD camera and Yokogawa CSU × 1 spinning disk confocal scan head with Spectral Aurora Borealis upgrade, four separate diode-pumped solid state laser lines (Spectral Applied Research, 405, 491, 561, and 642 nm). The images were analyzed using Volocity Software (Perkin-Elmer).

### Detection of ROS Using DHR123

The effect of salt on ROS production by neutrophils was assessed by DHR123 dye (Life Technologies). ROS oxidize DHR123 into R123, which emits a green fluorescence signal (36); thus, the amount of the green fluorescence signal indicates the amount of ROS production. Cells were preincubated with 25 µM of DHR123 dye for 15 min at 37°C. These preloaded cells were seeded into 96-well plates, and salt concentrations were altered by adding equal volumes of different RPMI media with either NaCl or choline chloride (109, 115, 121, 134, 159, 209, 309, and 509 mM). These cells were either unstimulated (−ve control) or activated by PMA or LPS. Fluorescence was measured at 10-min intervals for up to 60 min using an Omega fluorescence microplate reader. For the visualization and qualitative analysis, the same DHR123 plates were used for the confocal imaging after counter staining the nuclei with DAPI.

### Detection of ROS Using 2′,7′-Dichlorofluorescein Diacetate (DCFDA)

The effect of salt on ROS generation was confirmed by using a cell permeant reagent DCFDA (also known as H2DCFDA). This fluorogenic dye measures ROS activity within the cell. After diffusion into the cell, DCFDA/H2DCFDA is deacetylated by cellular esterases to a non-fluorescent compound, which is later oxidized by ROS into 2′,7′-dichlorofluorescein (DCF). DCF emits green fluorescence which is directly proportional to the amount of oxidized DCFDA to DCF. The maximum excitation and emission spectra are 495 and 529 nm, respectively (37, 38). Neutrophils were preloaded with 5 µM of DCFDA dye and either left unstimulated (−ve control) or activated by PMA at different salt and d-mannitol concentrations. Fluorescence was measured at 10 min intervals up to 60 min using an Omega fluorescence microplate reader as previously described.

### Confocal Imaging of Apoptosis

The neutrophils were prepared for immunostaining of apoptosis markers following the same procedure as described in the section titled “Immunofluorescence confocal imaging.” During immunofluorescence staining, anti-cleaved caspase-3 rabbit primary antibody (Asp175, Cell Signaling) at 1:1,000 dilution and goat anti-rabbit secondary antibody (Alexa Fluor 555, A21428, Life Technologies) at 1:1,000 dilutions were used. Further, these immunostained slides were imaged by confocal microscopy, as described previously.

### Bacterial Culture

A single colony of *Pseudomonas aeruginosa* (mPA01) and *Staphylococcus aureus* (RN4220) was picked from the respective agar plates and grown overnight in sterile LB-broth. The next day, the bacterial cultures were diluted 10-fold and further subcultured for three hours to eliminate the dead colonies or bacteria. Bacterial culture were harvested and washed three times in PBS (pH 7.4) by centrifugation at 5,000 *g* for 5 min at 4°C. After the third wash, the optical density (OD) of resuspension was determined at a wavelength of 600 nm. The bacterial concentration was determined by taking OD at 600 nm. The different MOI were determined by a formula (colony forming unit 10^8^ = OD600 × 30.88 − 99.607). NETosis was induced using either of these bacteria at an MOI of 20.

### Intracellular pH Measurement by SNARF Assay

SNARF^®^-4F 5-(and-6)-carboxylic acid (Thermo Fisher Scientific), dye was used to determine the intracellular pH changes in neutrophils. Carboxy SNARF-4F dye is typically used by exciting the dye at one wavelength (between 488 and 530 nm) while monitoring the fluorescence emission at two wave lengths (580 and 640 nm). The ratio of the fluorescence intensities of the two emission wavelengths 640/580 nm has been used as a proxy for intracellular pH (39). Neutrophils were preloaded with 10 µM dye for 15 min at 37°C and 5% (v/v) CO_2_. After the incubation, preloaded cells were washed and resuspended in fresh RPMI medium at the concentration of 1 × 10^6^ cells/ml. 50,000 neutrophils in a volume of 50 µL were seeded into a clear bottom black 96-well plate (BD Biosciences). An equal volume (50 µL) of RPMI containing different concentrations of the NaCl was added into the corresponding wells. These neutrophils were either left unstimulated (−ve control) or activated by PMA or LPS. Then, dual emission spectra of SNARF were measured. The changes in pH were recorded every 10 mins up to 90 min. The emission spectrum of SNARF undergoes a pH-dependent wavelength shift; therefore, the ratio (640/580 nm) of the fluorescence intensities from the dye at two emission wavelengths was used to determine the intracellular pH of neutrophils.

### Data Presentation and Statistical Analyses

Statistical analyses were performed using Graphpad Prism 7. Where applicable, One-way ANOVA with Dunnett’s and Tukey’s Post-tests, Two-way ANOVA with Bonferroni post-test, or *t*-test were used. A *p*-value of <0.05 was considered to represent a statistically significant difference. All data presented as mean ± SEM. The biological replicates and applied statistical tests are noted in the figure legend.

## Results

### Increasing NaCl Concentration Suppresses PMA-Mediated NETosis

**Figure 1:**
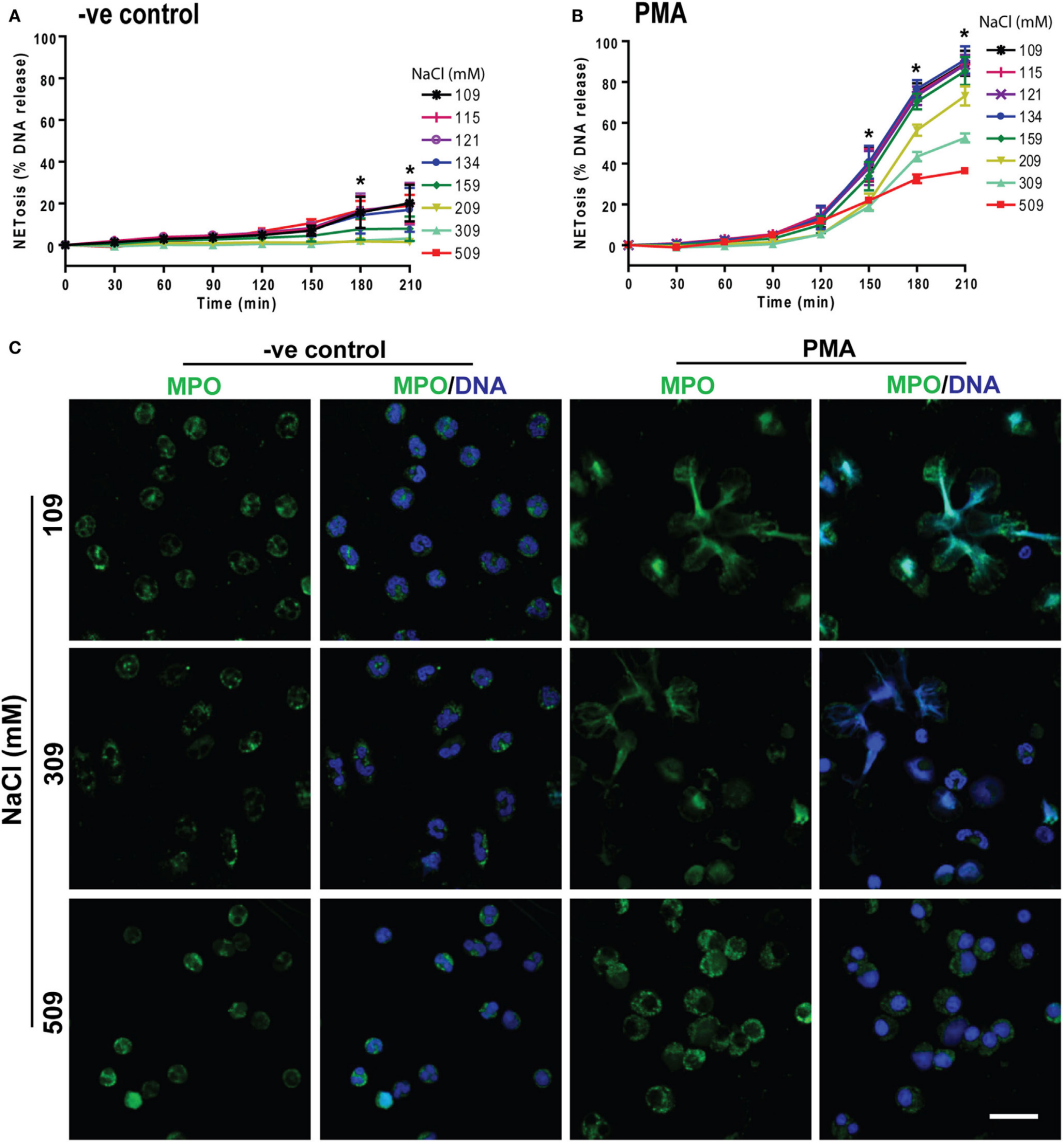
Increasing NaCl concentration suppresses phorbol myristate acetate (PMA)-mediated NETosis. NETosis assays were performed on human neutrophils resuspended in different NaCl concentrations (109, 115, 121, 134, 159, 209, 309, and 509 mM) with 5 µM Styox Green dye. These neutrophils were either unstimulated (−ve control) or activated or by PMA (25 nM). The Sytox Green florescence intensities (proxy for DNA release) were recorded by a plate reader every 30 min up to 210 min. **(A)** % DNA release (NETotic index) show less NETosis in higher salt conditions in unstimulated neutrophils (−ve control). **(B)** PMA treatment induces NETosis, and NaCl dose-dependently suppresses this form of NETosis. The suppression with NaCl concentrations at 309 mM and 509 mM is significant at several time points (*n* = 3−5, *p < 0.05; two-way ANOVA with Bonferroni’s multiple comparison post test). **(C)** Confocal microscopy of neutrophils with media only (−ve control) or PMA-treated in different salt conditions (109, 309, and 509 mM) were performed. Neutrophils were stained for DNA and myeloperoxidase (MPO); colocalization of the two signifies NETosis of neutrophils. Negative control neutrophils show no substantial neutrophil extracellular trap (NET) release, and higher NaCl conditions suppress the background NETosis. Drastic suppression of NETosis can be seen in PMA-treated neutrophils at 509 and 309 mM, but not in 109 mM salt conditions (*n* = 3−5; MPO-green; DNA-DAPI; scale bar, 22 µm). See Figure S1 in Supplementary Material for IgG isotype antibody and Figure S2 in Supplementary Material for more salt conditions, low magnification images.

To determine the effects of hypertonic saline (NaCl) on NETosis, we resuspended the neutrophils in RPMI medium with varying concentrations of NaCl (final concentrations of 109, 115, 121, 134, 159, 209, 309, and 509 mM) and 5 µM of the Sytox Green fluorescent dye. These neutrophils were either untreated (−ve control) or activated by NOX2-dependent prototypic agonist PMA. The fluorescence emission by Sytox Green was used as a proxy for extracellular DNA formation, and the green fluorescence signal was recorded every 30 mins up to 210 min. The kinetics of DNA release showed that changing NaCl concentration from 109 to 159 mM had little effect on NETosis. Hypertonic saline from 209 mM (1.2%, w/v) to 509 mM (3%, w/v) suppressed both background NETosis and PMA-induced NETosis (Figures 1A,B). Particularly, 309 mM (1.8%, w/v) and 509 mM (3%, w/v) NaCl concentrations significantly suppressed NETosis. To confirm true NETosis, confocal images were obtained after staining the DNA with DAPI and immunostaining MPO. Nuclear morphology and the colocalization of MPO (green) with DNA (blue) clearly indicated the suppression of NETosis in both unstimulated and PMA-activated neutrophils at hypertonic NaCl concentrations (Figure 1C). In unstimulated neutrophils, a few neutrophils underwent NETosis. However, with increasing concentrations of NaCl, the nuclei of neutrophils became condensed. When cells were activated with PMA in isotonic (or physiological) conditions, most cells underwent NETosis. However, at hypertonic conditions (especially at 309 and 509 mM NaCl), few or no neutrophils underwent NETosis. Rather, the nuclei became condensed. The isotype control for MPO immunostaining (Figure S1 in Supplementary Material) and low magnification images with all NaCl concentrations (Figure S2 in Supplementary Material) further confirmed the specificity of the antibodies and the effect of hypertonic saline on neutrophils.

### Increasing NaCl Concentration Suppresses PMA-Mediated ROS Production

**Figure 2:**
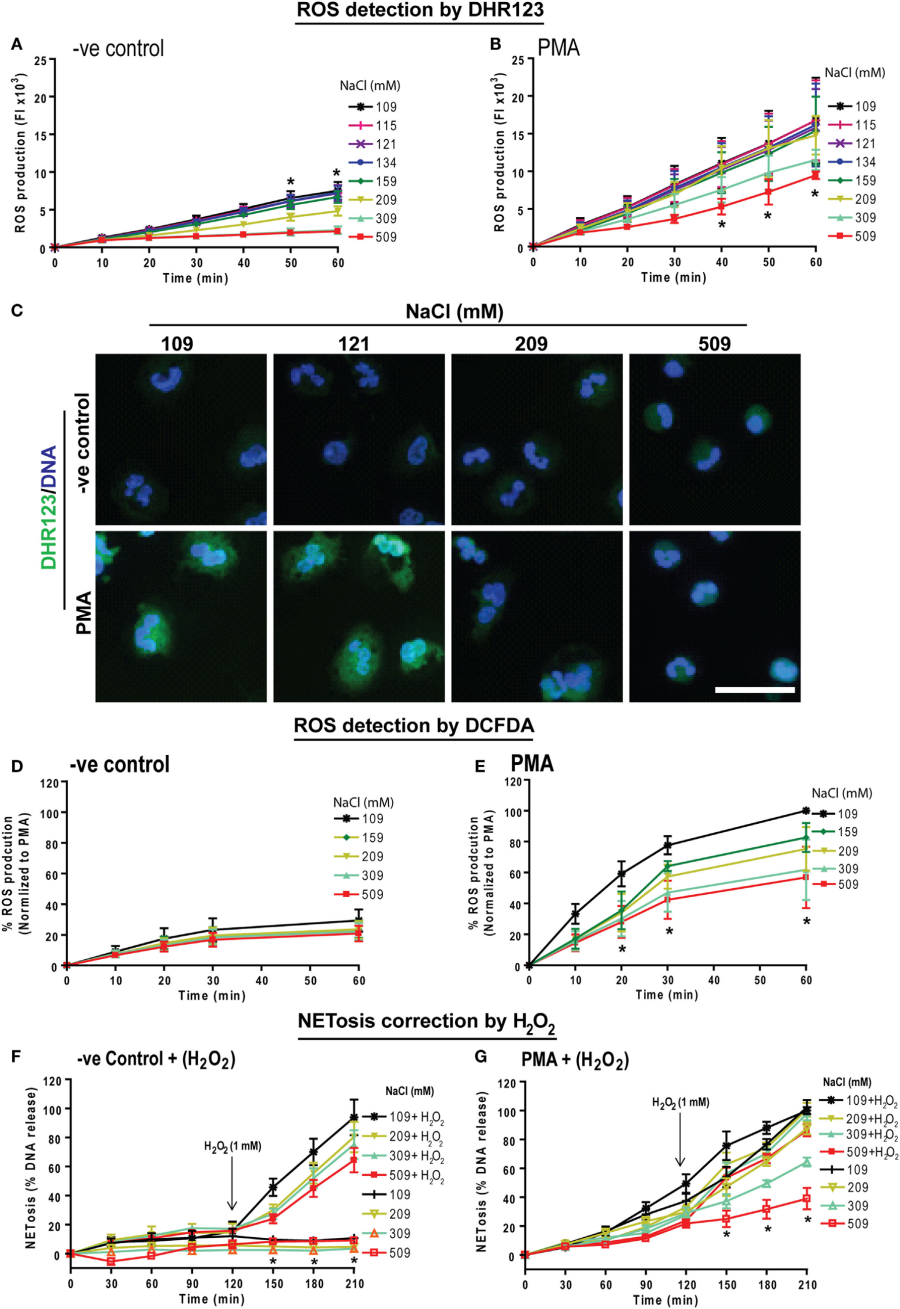
Increasing NaCl concentration suppresses phorbol myristate acetate (PMA)-mediated reactive oxygen species (ROS) production and hydrogen peroxide treatment restores NETosis inhibition. Human neutrophils were pretreated with cytosolic ROS indicator dye DHR123 or 2′,7′-dichlorofluorescein diacetate (DCFDA) in different NaCl concentrated media and were either unstimulated (−ve control) or activated by PMA (25 nM). ROS production kinetics was performed by plate reader assays up to 60 min. **(A)** Neutrophils in −ve control show a significant NaCl dose dependent suppression of background ROS generation. **(B)** ROS generation kinetics in PMA-treated neutrophils also show significant suppression of ROS in later time points for hypertonic saline conditions (309 and 509 mM) compared to the normotonic condition (109 mM). The suppression was statistically significant at time points 40, 50, and 60 min (*n* = 3−4; *p < 0.05; two-way ANOVA with Bonferroni’s multiple comparison posttest). **(C)** Neutrophils were stained for DNA and DHR123 to observe ROS production in cytosolic space. Confocal microscopy of DHR123-treated neutrophils with 109, 121, 209, and 509 mM NaCl, confirms a dose-dependent suppression of ROS production at higher concentrations in PMA-activated cells. R123 fluorescence was detected in both 109, and 121 mM but not in 209 and 509 mM NaCl condition. Unstimulated (−ve control) neutrophils show little or no ROS production (*n* = 3−4; R123-green; DNA, DAPI blue; scale bar 22 µm). **(D)** Intracellular ROS production measured using the DCFDA dye showed very minimal ROS production in −ve control, while **(E)** PMA treatment induced intracellular ROS production, which is dose dependently suppressed with higher NaCl concentration (*n* = 3; *p < 0.05; two-way ANOVA with Bonferroni’s multiple comparison posttest). See Figure S3 in Supplementary Material for more salt conditions and low magnification images. Furthermore, during NETosis, hydrogen peroxide was added to human neutrophils after 120 min exposure to different NaCl concentrations. **(F)** Hydrogen peroxide induced a significant amount of NETosis in a range of varying NaCl concentrations (109 – 509 mM). **(G)** In addition, Hydrogen peroxide rescued the suppression in PMA-induced NETosis in high salt concentrations, bringing the NETosis levels of neutrophils in 509 mM NaCl close to NETosis levels of neutrophils in 109 mM NaCl (*n* = 3; *p < 0.05; two-way ANOVA with Bonferroni’s multiple comparison post test).

NOX2-mediated ROS generation is important for PMA-mediated NETosis (25, 40, 41). Therefore, we investigated the effect of NaCl concentration on ROS production with a plate reader assay using the DHR123 dye. Neutrophils in media with different NaCl concentrations (109, 115, 121, 134, 159, 309, and 509 mM) were pre-loaded with DHR123 were either left untreated (−ve control) or activated usnig PMA. Changes in green fluorescence (oxidized DHR123 or R123) were recorded every 10 min up to 60 min. The % ROS production was calculated by considering the ROS produced by PMA-activated neutrophils under normotonic conditions at 60 min as 100%. DHR123 assays showed a small amount of background ROS production in unstimulated neutrophils. This baseline ROS production was suppressed by hypertonic saline. Above 209 mM NaCl, ROS production was significantly suppressed (Figure 2A). As expected, neutrophils stimulated with PMA generated much higher amounts of ROS under normotonic conditions (Figure 2B). However, beyond 109 mM of NaCl, ROS production was significantly suppressed. Images of neutrophils stained with DHR123 and DAPI confirmed the ROS production under normotonic conditions, and the suppression of ROS production at hypertonic NaCl concentrations (Figure 2C). The low magnification images of neutrophils stained with DHR123 and DAPI for all eight salt concentrations (Figure S3 in Supplementary Material) showed the dose-dependent suppression of ROS production.

We further validated the effect of increasing NaCl concentration on ROS production in neutrophils using another dye, DCFDA (also known as H2DCFDA). DCFDA is oxidized by ROS into DCF, which emits green fluorescence. Neutrophils were preloaded with 5 µM DCFDA and were left untreated or activated by PMA at different NaCl concentrations. Fluorescence was measured every 10 mins up to 60 min. Similar to the results observed by DHR123 assay, DCFDA assays showed that hypertonic NaCl suppressed ROS production (Figures 2D,E). Therefore, hypertonic NaCl suppresses ROS production in neutrophils.

### H_2_O_2_ Rescues Hypertonic NaCl-Mediated Suppression of NETosis

To determine whether providing oxidants to neutrophils could overcome the hypertonic NaCl-mediated suppression of ROS production, we used H_2_O_2_, a key oxidant generated by neutrophils to execute NOX2-dependent NETosis (42, 43). We have previously shown that 1 mM H_2_O_2_ induces NETosis (20). Therefore, we induced NOX2-dependent NETosis using PMA in neutrophils treated with different NaCl concentrations (109, 209, 309, 509 mM) and monitored DNA release using Sytox Green assay. After 120 min, 1 mM H_2_O_2_ was added and NETosis kinetics was monitored up to 210 min. Data showed that H_2_O_2_ treatment increased NET release by reversing the inhibitory effect of hypertonic NaCl on NETosis (Figures 2F,G). Collectively, these data (Figures 1 and 2) suggest that hypertonic NaCl suppresses NET formation by suppressing ROS production during NOX2-dependent NETosis.

### Increasing Choline Chloride Concentration Suppresses PMA-Mediated ROS Production and NETosis

**Figure 3:**
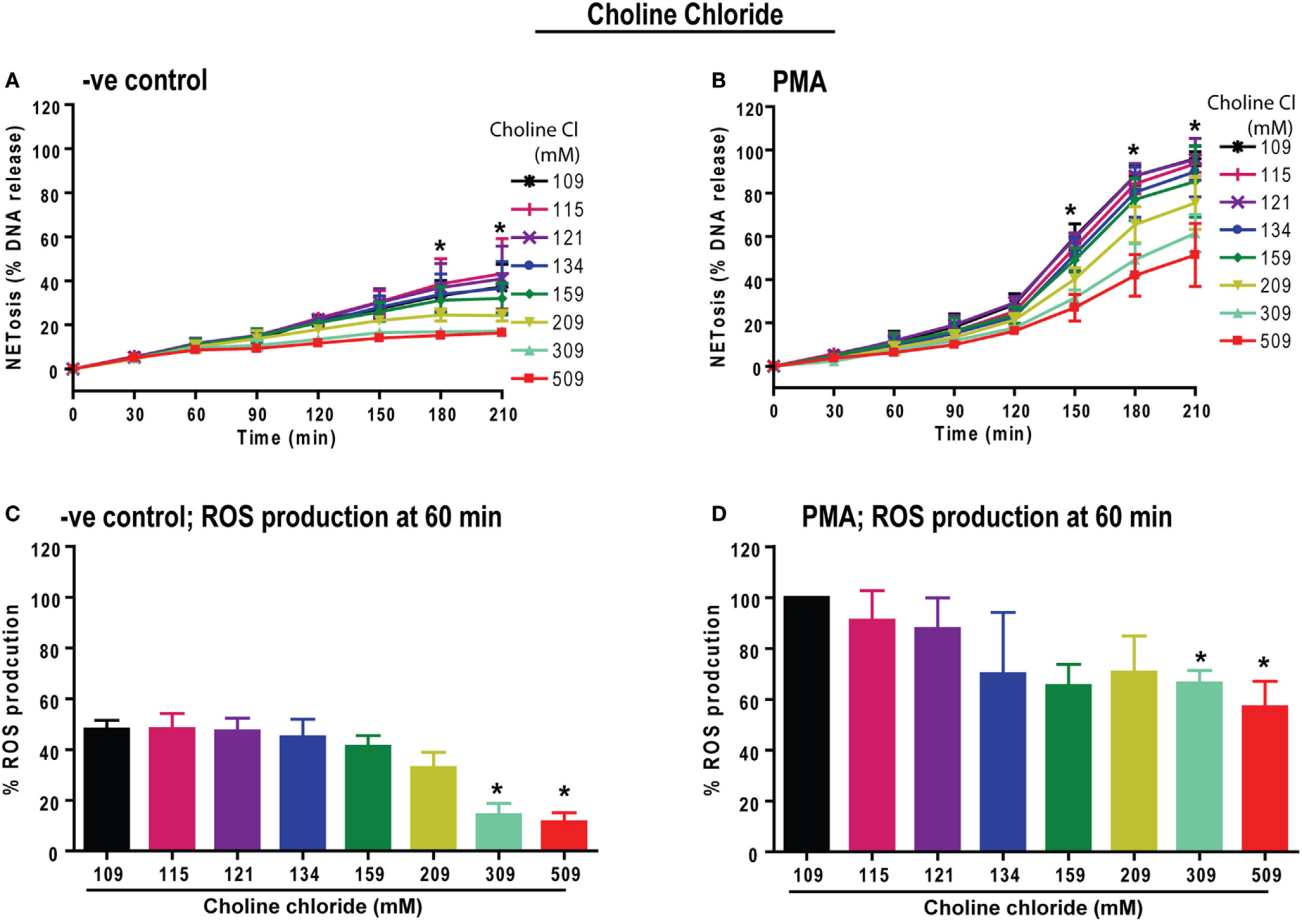
Increasing choline chloride suppresses phorbol myristate acetate (PMA)-mediated reactive oxygen species (ROS) production and neutrophil extracellular traps (NETs) release. Neutrophils were resuspended in equimolar choline chloride (ChlCl) containing RPMI media, substituting different concentrations of NaCl. These neutrophils were used for the Sytox Green NETosis and DHR123-based ROS production kinetics by plate reader assays. **(A)** −ve control neutrophils treated with ChlCl shows dose-dependent suppression of background % DNA release at 309 and 509 mM ChlCl conditions. **(B)** PMA-treated neutrophils show significant dose-dependent suppression of NET release at 309 and 509 mM ChlCl conditions at time points 150, 180 and 210 min (*n* = 3; two-way ANOVA with Bonferroni’s multiple comparison post test). **(C)** The % ROS production was calculated by considering the PMA-mediated ROS production at normotonic condition as 100% during 60-min time point. At 60 min, background ROS production is suppressed by high ChlCl doses of 309 and 509 mM. **(D)** PMA-treated neutrophils experience significant suppression of ROS production at the 60-min time point (*p < 0.05; one-sample *t*-test compared to hypothetical value 100).

Next, we determined whether the sodium or chloride ion in the compound NaCl is responsible for the suppression of ROS and NOX2-dependent NETosis. To test this, we substituted choline chloride for sodium chloride and performed NETosis experiments. The NETosis kinetics elucidated that increasing concentrations of choline chloride has the same overall effect as increasing the concentration of NaCl; choline chloride suppresses both baseline and PMA-induced NETosis (Figures 3A,B). In addition, the % ROS kinetics showed suppression of baseline and PMA-induced ROS production with increasing choline chloride concentrations (Figures 3C,D). Therefore, an increase in Na^+^ ion concentration may not be responsible for the effect of hypertonic saline on NETosis kinetics. Since an increase in equimolar concentrations of NaCl and choline chloride have comparable effects, it becomes clear that hypertonic saline-mediated suppression of NETosis could depend on the increase in chloride concentrations or osmolarity.

### Increasing Osmolarity by Non-Ionic Osmolytes Suppresses PMA-Mediated ROS Production and NETosis

**Figure 4:**
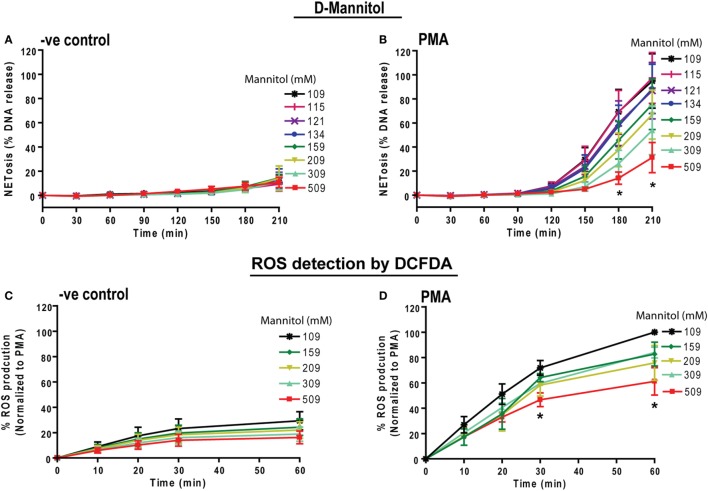
Increasing concentrations of d-mannitol suppress phorbol myristate acetate (PMA)-mediated reactive oxygen species (ROS) production and NETosis. Human neutrophils were treated with d-mannitol and d-sorbitol to determine the effects of osmolarity during NETosis. **(A)** Unstimulated neutrophils treated with d-mannitol showed no suppression in background NETosis. **(B)** In contrast, PMA-treated cells in different concentrations of d-mannitol showed significantly lower NETosis in a dosage-dependent manner (*n* = 3, *p < 0.05; two-way ANOVA with Bonferroni’s multiple comparison post test). ROS production was measured by using neutrophils preloaded with DCFDA under different d-mannitol concentrations. **(C,D)** The ROS production in PMA-activated neutrophils is suppressed by high d-mannitol concentrations (*n* = 3, *p < 0.05; two-way ANOVA with Bonferroni’s multiple comparison post test). Similar to d-mannitol, d-sorbitol treatment did not alter background NETosis activation in −ve control while it suppresses PMA-induced NETosis. See Figure S4 in Supplementary Material for d-sorbitol data.

To determine whether osmolarity is a key factor in regulating PMA-mediated NETosis, we performed experiments in the presence of equimolar concentrations of non-ionic osmolytes such as d-mannitol and d-sorbitol. Neutrophils resuspended in RPMI media containing different concentrations of d-mannitol or d-sorbitol (109, 115, 121, 134, 159, 209, 309, and 509 mM) were either unstimulated (−ve control) or activated by PMA. The % DNA release kinetics showed that both d-mannitol (Figures 4A,B) and d-sorbitol (Figure S4 in Supplementary Material) dose-dependently suppressed PMA-mediated NETosis. Determining ROS production by DCFDA plate reader assays showed that increasing concentrations of d-mannitol also suppressed ROS production (Figures 4C,D). Collectively, these data suggest that increased osmolarity suppresses ROS production and subsequently suppresses NETosis.

### Increasing NaCl Concentrations Suppresses LPS-Mediated ROS Production and NETosis

**Figure 5:**
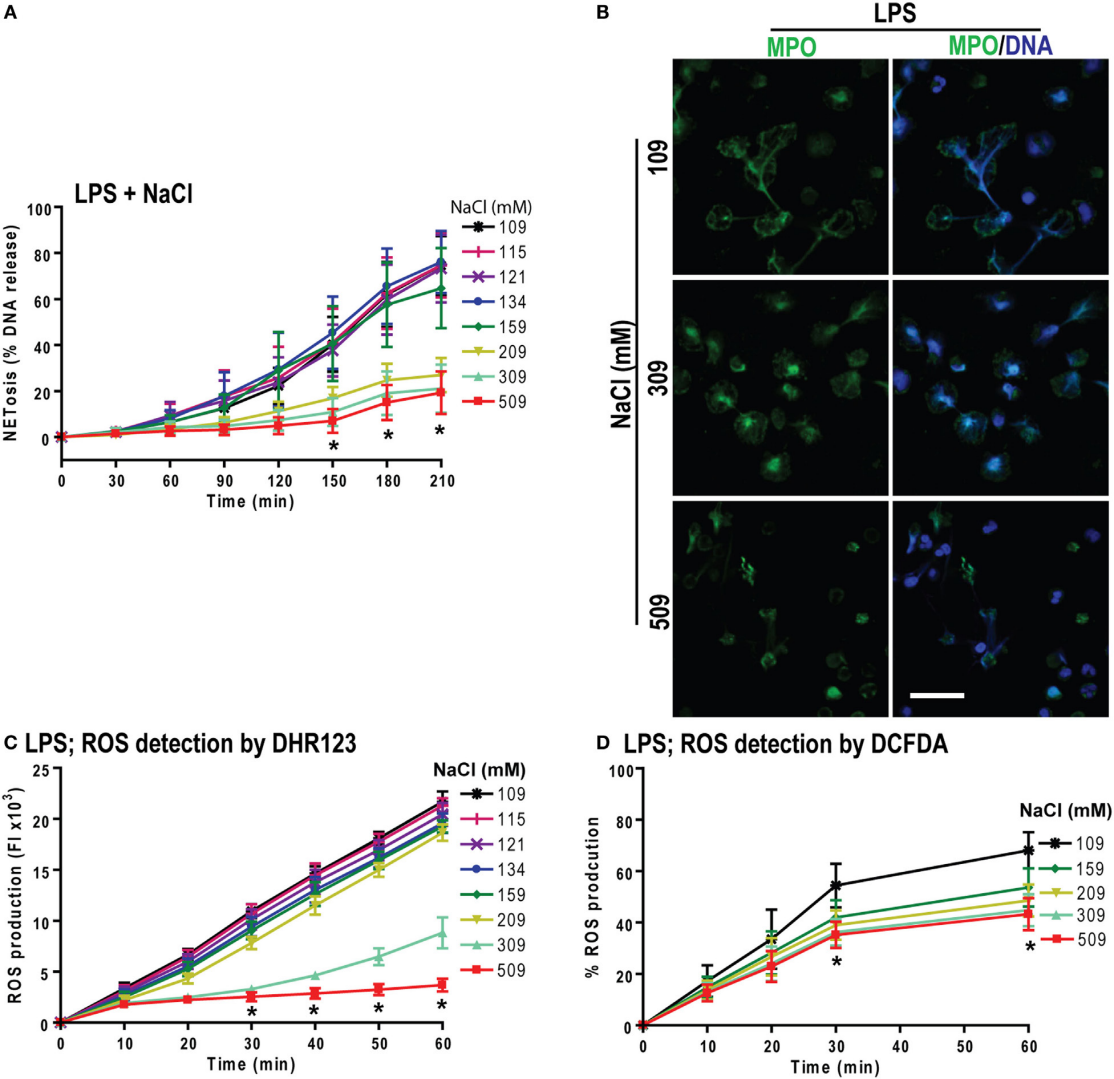
Increasing NaCl concentration suppresses lipopolysaccharide (LPS)-mediated reactive oxygen species (ROS) production and NETosis. **(A)** % total DNA release (NETosis) of LPS-treated neutrophils are significantly suppressed at higher NaCl concentrations. The suppression is significant in high salt concentrations (209, 309, and 509 mM) at various time points (*n* = 3−5; *p < 0.05; two-way ANOVA with Bonferroni’s multiple comparison post test). **(B)** Confocal microscopy of neutrophils shows significant LPS-induced NETosis suppression at hypertonic saline concentrations compared to lower salt dose treatments (*n* = 3−4; myeloperoxidase, green; DNA, DAPI blue; scale bar, 22 µm). **(C,D)** LPS-induced ROS production measured by DHR123 and DCFDA dyes, using fluorescent plate reader show the ROS suppression in LPS-treated neutrophils at high NaCl concentrations over 60 min (*n* = 3−5; *p < 0.05; two-way ANOVA with Bonferroni’s multiple comparison post test).

Both PMA and LPS (*E. coli* strain 0111:B4; O128:B12) induce NOX2-dependent NETosis (25, 44). Therefore, we tested the effect of increasing NaCl concentrations in LPS-mediated NETosis. The NETosis kinetics as determined by the % DNA release showed a NaCl concentration-dependent suppression of LPS-mediated NETosis (Figure 5A). Confocal microscopy images of DAPI and MPO-immunostained neutrophils confirmed the suppression of NETosis at higher NaCl concentrations (Figure 5B).

To determine whether hypertonic NaCl also attenuates LPS-mediated ROS production, we determined ROS production in neutrophils by DHR123 and DCFDA assays. Hypertonic NaCl also suppressed ROS production in a dose-dependent manner (Figures 5C,D). Therefore, hypertonic NaCl suppresses LPS-mediated ROS production and subsequent NOX2-dependent NETosis.

### Increasing Osmolarity Suppresses LPS-Mediated ROS Production and NETosis

**Figure 6:**
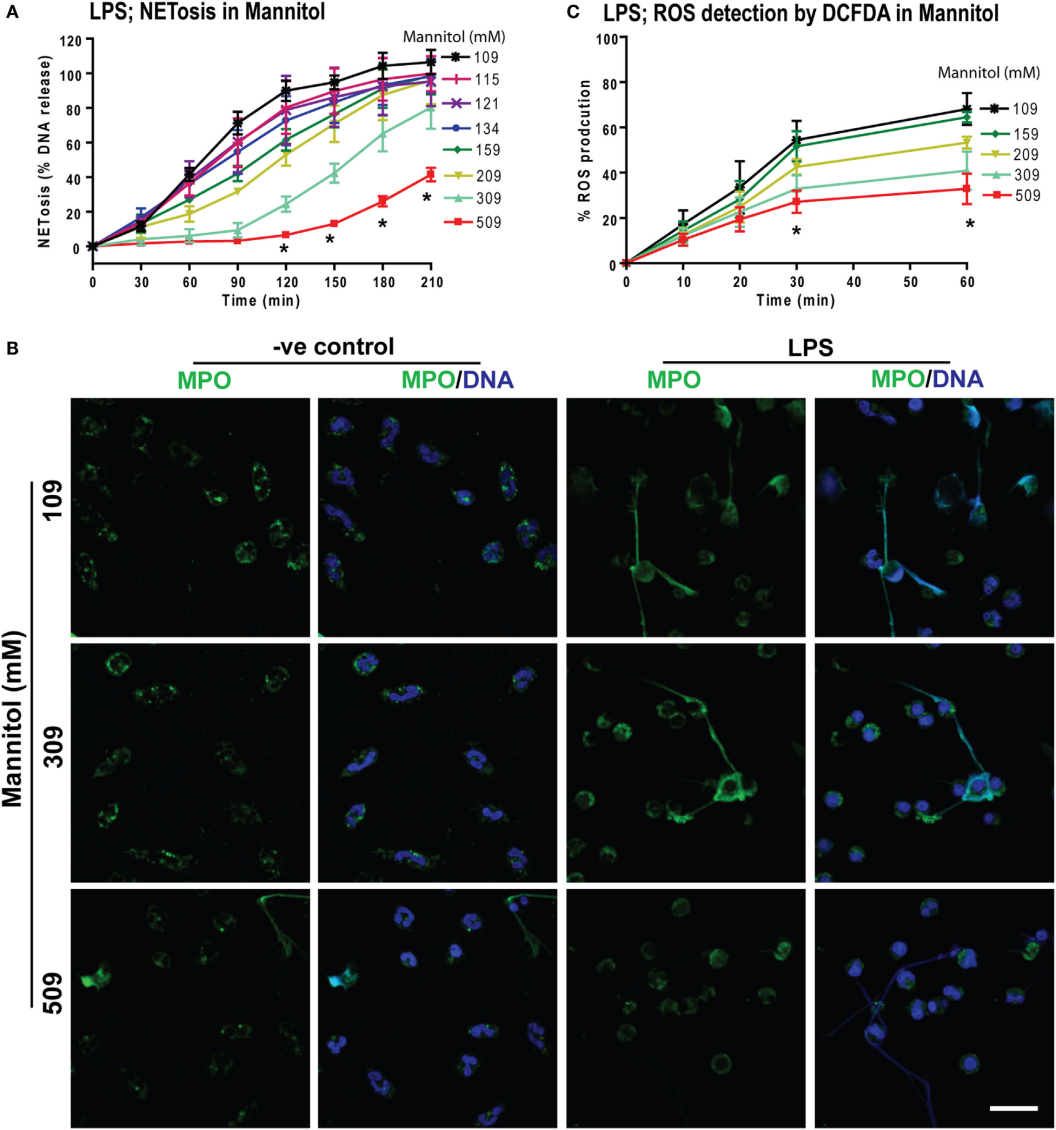
Increasing concentrations of d-mannitol suppress lipopolysaccharide (LPS)-mediated reactive oxygen species (ROS) production and NETosis. **(A)** The % DNA release NETosis kinetics shows the suppression of LPS-mediated NETosis in increasing concentrations of d-mannitol (*n* = 3; *p < 0.05; two-way ANOVA with Bonferroni’s multiple comparison post test). **(B)** Myeloperoxidase-immunostained and confocal images of unstimulated neutrophils (−ve control) and LPS-treated cells in different concentrations of d-mannitol shows the suppression of NETosis at higher concentration (*n* = 3; MPO-green; DNA-DAPI; scale bar, 22 µm). **(C)** LPS-induced ROS production measured by DCFDA in neutrophils under different d-mannitol concentrations, show the ROS suppression at high d-mannitol concentrations over 60 min (*n* = 3; *p < 0.05; two-way ANOVA with Bonferroni’s multiple comparison post test). See Figures S5 and S6 in Supplementary Material for d-sorbitol NETosis and ROS production data.

To determine whether increasing osmolarity suppressed LPS-mediated ROS production and NETosis, we used the osmolytes d-mannitol and d-sorbitol. The % DNA release was suppressed at higher concentrations of d-mannitol (Figure 6A) and d-sorbitol (Figure S5 in Supplementary Material) in a concentration-dependent manner. Untreated or LPS-activated neutrophils in different d-mannitol concentrations were stained for DNA (DAPI) and immunostained for MPO. Images showed colocalization of MPO and DNA, which indicated LPS-induced NET formation. Increasing concentrations of d-mannitol also suppressed NETosis (Figure 6B).

Measuring ROS production by DCFDA assays showed that d-mannitol suppressed LPS-mediated ROS production in a concentration-dependent manner (Figure 6C). Choline chloride also suppressed LPS-mediated ROS production in a concentration-dependent manner (Figure S6 in Supplementary Material). These data suggest that increased osmolarity suppresses LPS-mediated NETosis in a dosage dependent manner through the suppression of ROS production. Collectively (Figures 1–6), an increased concentration of neither sodium nor chloride was solely responsible for the suppressive effect of hypertonic NaCl on ROS production. Increased osmolarity was sufficient to suppress ROS production and subsequent NOX2-mediated NETosis.

### Hypertonic NaCl Does Not Alter Intracellular pH of Neutrophils

We and other have shown that intracellular pH is an important factor in regulating NOX2-dependent NETosis (45–47). Therefore, to verify whether hypertonic NaCl alters cellular pH, we measured the intracellular pH of neutrophils that were either untreated (−ve control) or activated by PMA or LPS in different NaCl conditions (109, 159, 209, 309, 509 mM) in pH preadjusted media (6.6 and 7.8). Ratios of SNARF duel wavelength measurements indicated that pH standard at pH 7.8 and 6.6 showed higher and lower ratios, respectively. These results reflect that the assay worked as expected. However, hypertonic saline conditions did not significantly change intracellular pH (Figure S7 in Supplementary Material). Therefore, the effect of hypertonic NaCl on suppressing NOX2-mediated NETosis does not occur by altering intracellular pH of the neutrophils.

### Increasing NaCl Switches NOX2-Dependent NETosis to Apoptosis

**Figure 7:**
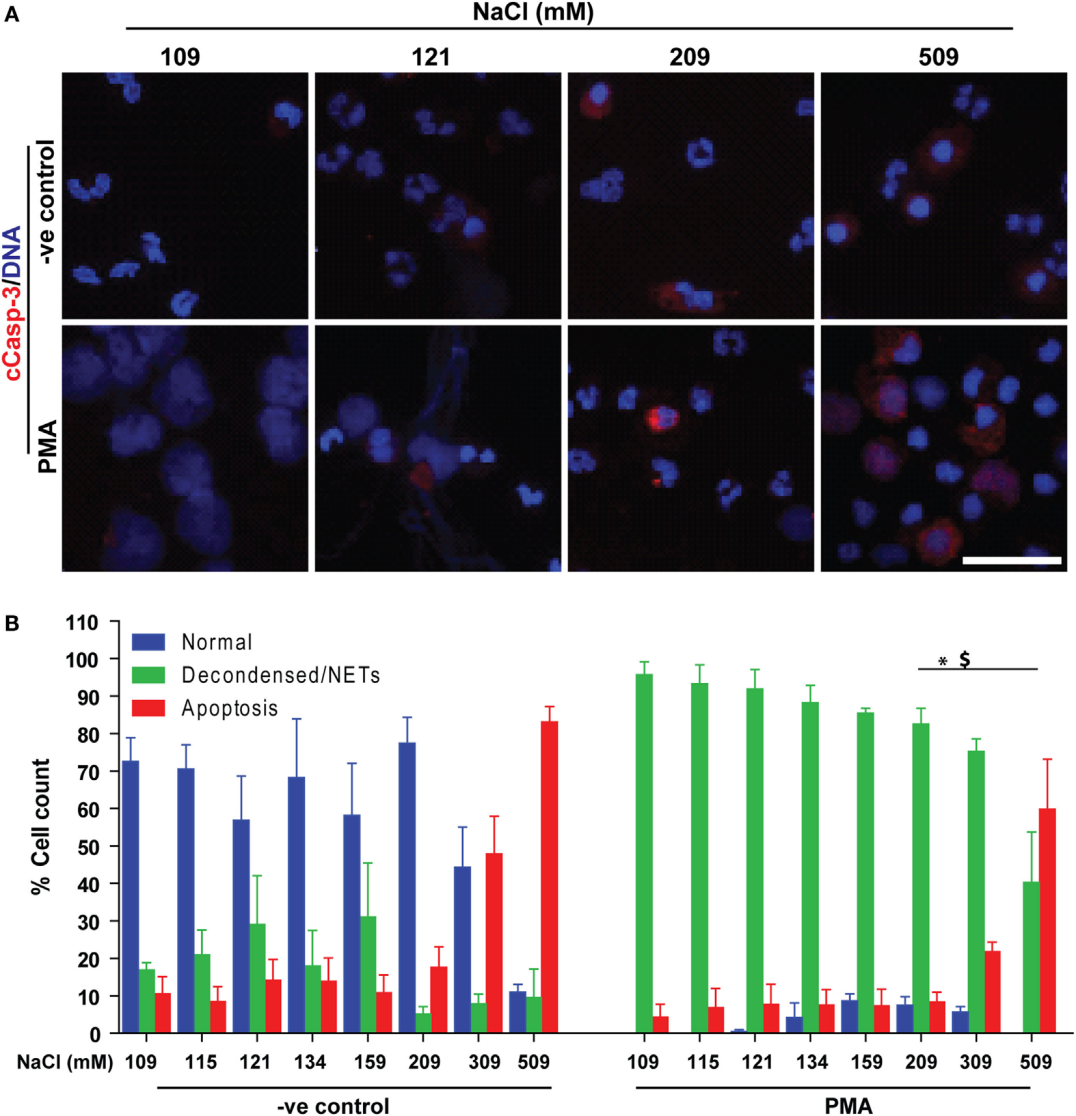
Increasing NaCl switches phorbol myristate acetate (PMA)-mediated NETosis to apoptosis. Human neutrophils were either unstimulated (−ve control) or activated by PMA, and immunostained with DAPI (DNA) and cCasp-3 in different salt concentration conditions (109, 115, 121, 134, 159, 209, 309, and 509 mM). **(A)** Confocal microscopy of unstimulated (−ve control) neutrophils show few apoptotic neutrophils at higher NaCl concentrations, but not lower ones. PMA-treated neutrophils with normotonic NaCl treatment undergo complete NETosis. During the hypertonic conditions, NETosis is suppressed, and the cells appear apoptotic (*n* = 3; cCasp-3; red, DNA; DAPI, scale bar, 22 µm). **(B)** % total cell count (based on the immunostaining and cellular morphology) data show a clear trend of NETosis suppression during unstimulated and PMA-mediated activation of neutrophils, while apoptosis is promoted with increasing concentration of NaCl. Collectively, data indicate that suppression of NETosis leads to apoptosis at high salt treatments (*n* = 3; *p < 0.05; one-way ANOVA with Tukey’s multiple comparison post test). See Figure S8 in Supplementary Material for low magnification images.

Since neutrophils were not dying by NETosis at hypertonic NaCl concentrations, we examined whether these cells were dying *via* apoptosis. We conducted immunocytochemistry experiments to determine two key apoptotic makers, cleaved caspase-3 (cCasp-3) and condensed nuclei. The immunostaining experiments were performed after treating neutrophils (untreated or PMA-treated) with varying concentrations of NaCl for 210 min. Qualitative examination of nuclear morphology and cCasp-3 staining showed that higher salt concentrations suppressed NETosis by promoting apoptosis in unstimulated (−ve control) and PMA-activated neutrophils (Figure 7A). Quantitative analysis of the % total cell counts (based on the nuclear morphology, DNA decondensation, NETs release and cCasp-3 staining) confirmed the suppression of NETosis and promotion of apoptosis (Figure 7B). Hence, this dataset suggests that suppressing NOX2-dependent NETosis by hyperosmotic NaCl is associated with an increase in apoptosis.

### Effect of Increasing NaCl Concentration on NOX2-Independent NETosis

**Figure 8:**
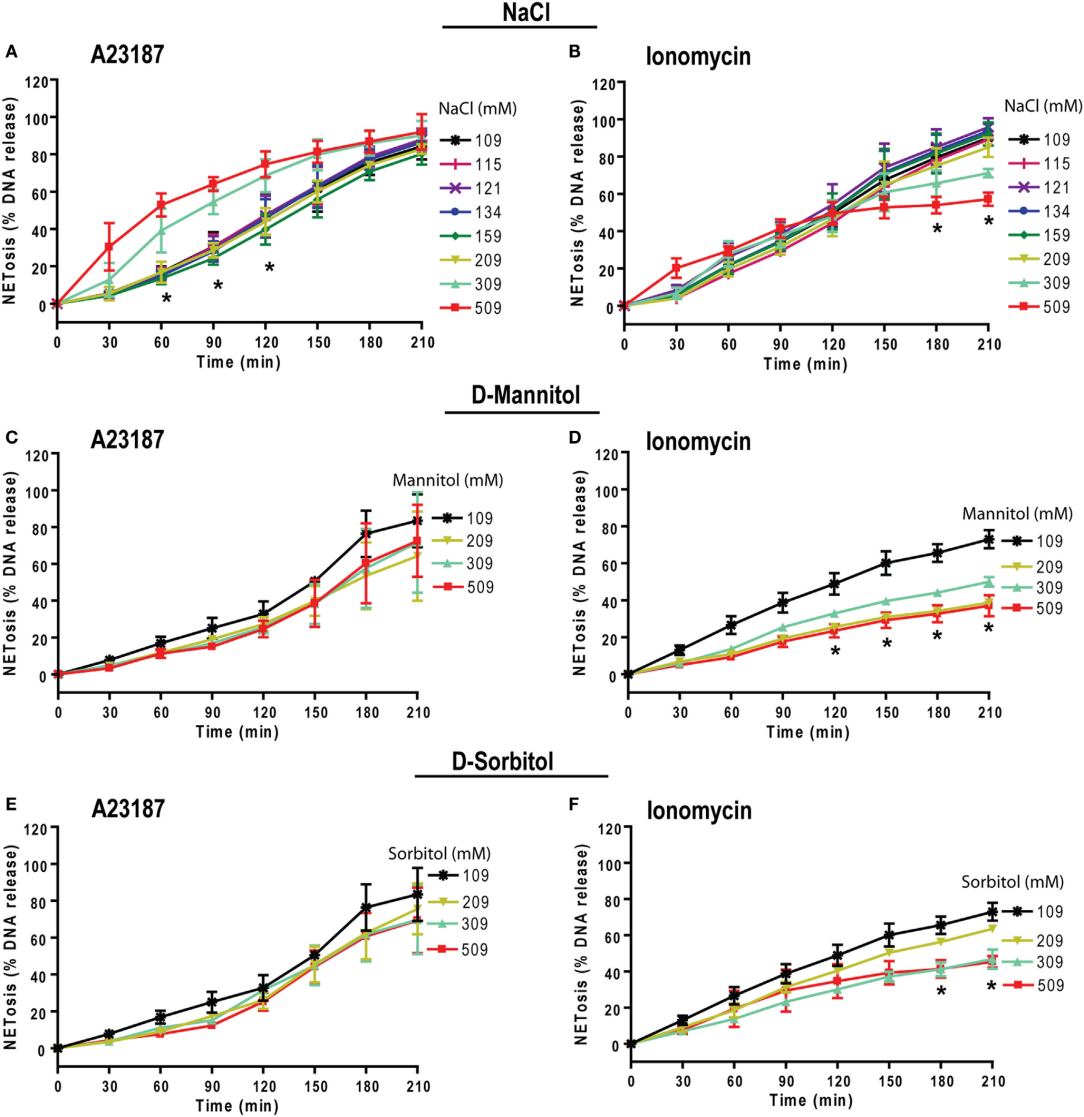
Increasing NaCl and osmolytes concentration suppresses ionomycin-induced NETosis. Human neutrophils in different osmolytes (NaCl, d-mannitol, and d-sorbitol) were treated with Nox-independent NETosis agonists A23187 and ionomycin. **(A)** Initial kinetics of the neutrophils under high concentration showed more NETosis. High NaCl concentrations were not able to suppress NETosis in response to A23187; however, this effect was lost by later time points leading to no overall difference by 210 min. **(B)** In contrast, ionomycin-induced NETosis show some suppression at later time points. **(C,D)** Neutrophils treated with different d-mannitol concentrations showed NETosis suppression only in ionomycin activated neutrophils. **(E,F)** Neutrophils treated under d-sorbitol conditions also show the same suppression pattern as of **(C,D)** (*n* = 3; Two-way ANOVA with Bonferroni’s multiple comparison post test).

Neutrophils could also undergo NOX2-independent NETosis. As previously discussed, calcium ionophores A23187 and ionomycininduce this type of NETosis (19, 21, 24, 48, 49). To determine the effect of higher NaCl concentration on NOX2-independent NETosis, we incubated neutrophils with A23187 or ionomycin (obtained from *S. conglobatus*) under different NaCl concentrations. Unlike NOX2-dependent NETosis, the suppressive effect of high NaCl concentrations on NOX2-independent NETosis was not clear. The effect of hypertonic saline was different at different time points and varied based on the agonist used. For A23187-induced NETosis, hypertonic saline increased NETosis at the early time points but not at later time points. In contrast, for ionomycin-induced NETosis, hypotonic saline suppressed NETosis at the later time points (Figures 8A,B). Furthermore, NaCl was replaced by d-mannitol or d-sorbitol to study the effect of osmolarity during NETosis mediated by A23187 and ionomycin. The Sytox green kinetics showed suppression of NETosis only for ionomycin condition at higher concentrations of d-mannitol (Figures 8C,D) and d-sorbitol (Figures 8E,F). Collectively, the NETosis kinetics data show that hypertonic saline and high concentrations of osmolytes suppressed ionomycin-mediated NETosis, but not A23187-mediated NETosis. Inconsistent effects of NaCl, d-mannitol, and d-sorbitol on calcium-ionophore-induced NETosis suggest that these molecules alter NOX2-independent NETosis in a complex manner.

### Increasing NaCl Concentrations Suppresses *Pseudomonas aeruginosa*- But Not *Staphylococcus aureus*-induced NETosis

**Figure 9:**
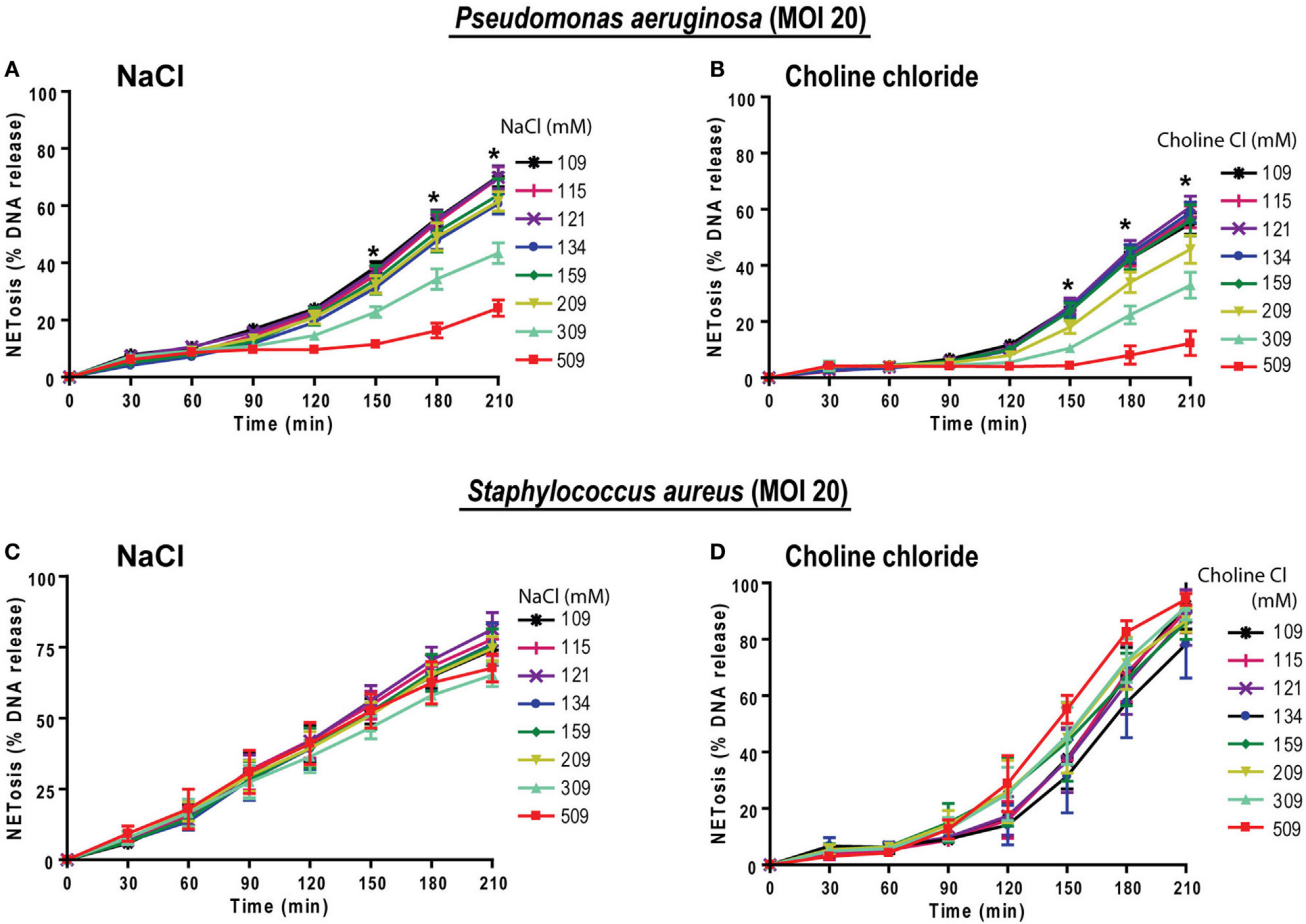
Increasing NaCl concentrations suppresses *Pseudomonas aeruginosa*-mediated NETosis. Neutrophils in different NaCl and choline chloride concentrations were activated either with *P. aeruginosa* or *S. aureus* (multiplicity of infection 20). **(A,B)** The % total NETosis induced by *P. aeruginosa* is suppressed during high NaCl or choline chloride concentrations compared to normal treatments (109 mM). **(C,D)** In contrast to *P. aeruginosa*-induced NETosis, *S. aureus*-induced NETosis was not suppressed by NaCl or choline chloride addition over 210 min (*n* = 3; *p < 0.05; two-way ANOVA with Bonferroni’s multiple comparison post test).

To examine the relevance of hypertonic saline in bacteria-induced NETosis, we cultured neutrophils with NETosis-inducing ratios (MOI of 20) of Gram-negative (*P. aeruginosa*) and Gram-positive (*S. aureus*) bacteria in different NaCl and choline chloride concentrations. Under normotonic conditions the % DNA release analyses showed that both of these bacteria induced NETosis at 20 MOI. Increasing concentration of both NaCl and choline chloride suppressed *P. aeruginosa*-mediated NETosis (Figures 9A,B). This pattern becomes evident beyond 209 mM of NaCl or choline chloride concentrations. However, neither NaCl nor choline chloride suppressed *S. aureus*-induced NETosis (Figures 9C,D). Therefore, hypertonic saline could differentially regulate different bacterial infections.

## Discussion

Hypertonic saline is used for treating a number of different diseases, including NETosis-associated CF and sepsis (50). Hypertonic saline treatment improves mucociliary clearance, reduces pulmonary exacerbations, and improves the quality of life in CF patients (51). Although multiple mechanisms have been proposed, the underlying mechanism of this therapy is still not completely understood. Currently, it is well established that neutrophil infiltration and excessive NETosis leads to disease exacerbation in CF patients. In this study, we investigated the regulatory mechanism of hypertonic saline on NETosis. We found that hypertonic saline that is commonly used in treatments (3% saline or 509 mM) suppresses NOX2-dependent NETosis induced by LPS and PMA. In addition, the suppressive effect of hypertonic saline on NETosis was partially regulated by suppressing ROS production, which is primarily exerted by an increase in osmolarity. At hypertonic saline condition, neutrophils undergo apoptosis instead of NETosis. The effect of hypertonic saline on NOX2-independent NETosis does not follow the same kinetics as of NOX2-dependent NETosis. The effect of hypertonic saline in suppressing bacteria-induced NETosis was also different depending on the type of bacteria. While hypertonic saline suppresses *P. aeruginosa*-induced NETosis, it does not alter *S. aureus-*induced NETosis. Therefore, hypertonic saline is likely to exert differential effects on neutrophil death based on NETosis-inducing agonists and bacteria. For example, different bacteria predominate different diseases and different stages of the lung disease in patients with CF. Therefore, hypertonic saline could have differential effect at different stages and disease conditions.

Prototypic agonists PMA (20, 25, 52, 53) and Gram-negative cell wall component LPS (*E. coli* strain 0111:B4; O128:B12) (25, 44, 54) are known to induce NOX2-dependent NETosis. Our studies show that hypertonic saline suppresses NETosis induced by both PMA and LPS (Figures 1 and 5; Figures S1, S2, and S4 in Supplementary Material). Replacing NaCl with choline chloride and non-ionic osmolytes such as d-mannitol and d-sorbitol also suppresses PMA- and LPS-mediated NETosis (Figures 3 and 6; Figure S5 in Supplementary Material). Choline chloride, made of the choline cation and chloride anion (55), results in similar suppression of NETosis as observed through NaCl (Figures 1 and 3). Therefore, an increase in sodium ion concentration is not sufficient to suppress NETosis. d-mannitol and d-sorbitol, 6-carbon sugar-related molecules, are known to dehydrate cells by osmotic action (56, 57). These osmolytes in equimolar concentrations exert similar suppressive effects on PMA- and LPS-mediated NETosis (Figures 3 and 6; Figures S4 and S5 in Supplementary Material). Therefore, increased osmolarity of these solutions is a key factor in suppressing NETosis. Gram-negative bacteria such as *P. aeruginosa* (58) and Gram-positive bacteria such as *S. aureus* (16, 19, 54) also induce NOX2-dependent NETosis. Hypertonic saline and choline chloride suppressed *P. aeruginosa*, but not *S. aureus-*induced NETosis (Figures 9A–D). Therefore, both ROS production and others factors such as bacterial cell wall are likely responsible for altering hypertonic saline-mediated regulation of NETosis.

Reactive oxygen species generation is a key factor for inducing LPS- and PMA-mediated NETosis (25, 40, 41, 59). Hypertonic NaCl (Figures 2 and 5; Figure S3 in Supplementary Material), choline chloride (Figure 3; Figure S6 in Supplementary Material), and osmolytes (Figures 4 and 6; Figures S4 and S5 in Supplementary Material) all suppress PMA- and LPS-mediated ROS production. Reduction in water content of the cells could affect several cellular processes (60, 61). Therefore, the suppressive effect of these solutes and osmolytes can partly be exerted by the reduction in ROS production. During NOX2-dependent NETosis, superoxide generated by NADPH oxidase is subsequently converted to H_2_O_2_, which is converted to HOCl by MPO in the presence of chloride ions (40, 62–64). Our studies and other reports show that 1 mM H_2_O_2_ induces NETosis in neutrophils (20, 40, 65). Supplementing 1 mM H_2_O_2_ to neutrophils rescues the suppressive effect of hypertonic saline (Figures 2F,G). Therefore, the supressive effect of hypertonic saline is at least partly due to the suppression of ROS production in these cells.

Many groups including ours have studied the intracellular pH change during NETosis. Maueröder et al. and our group have shown that increasing pH increases both NOX2-dependent and -independent NETosis by increasing ROS production, mitochondrial ROS, histone cleavage and citrullination of H3 in respective pathways of NETosis (45–47). However, hypertonic conditions did not alter intracellular pH. Therefore, pH is not a factor in regulating hypertonic saline induced alterations in neutrophil death.

It is important to understand the fate of neutrophils during hypertonic saline-induced suppression of NETosis. Interestingly, hypertonic saline suppresses NETosis while promoting apoptotic cell death in neutrophils (Figure 7; Figure S8 in Supplementary Material). In the context of inflammation, anti-inflammatory apoptotic neutrophil death could promote the resolution of inflammation (66). Neutrophils are recruited from the circulation to participate in lung immunity, in which they kill bacteria via either phagocytosis or NETosis (67, 68). Minimizing unwanted NETosis helps avoid the presence of injurious NETotic/cytoplasmic contents in the airways. The lifespan of neutrophils is limited, and these cells normally undergo apoptosis within 7−24 h. This thereby facilitates the clearance of neutrophils from inflammatory sites (69). Hypertonic saline switches the pathways of neutrophil death from NETosis to apoptosis. Thus, it could help to effectively clear neutrophils from the inflamed tissues.

Hypertonic saline and osmolytes did not have similar effect on calcium-induced NETosis (Figure 8). Calcium ionophores induce hypercitrullination and a rapid form of NETosis (19, 21, 24, 48, 49). The fact that increasing NaCl and osmolarity increases A23187-induced NETosis, particularly at early time points suggests that hypertonic conditions could facilitate neutrophil death when the cells rapidly die by NOX2-independent NETosis. Although A23187 and ionomycin are calcium ionophores, NET induction by these two molecules are differentially regulated by various factors and their mode of action is not identical (19, 24, 48, 70). In 2007, Sara et al. demonstrated that changes in chloride ion modulate neutrophil Ca^2+^ homeostasis by regulating Ca^2+^ release from intracellular stores, and degranulation (71). The ability of the chloride ion to increase intracellular calcium may explain why hypertonic saline transiently increases in A23187-mediated NETosis. However, the regulatory mechanism of ionomycin-mediated NETosis may be different. Some of these differences could be responsible for the differences in the inhibitory effects of hypertonic saline and osmolytes on A23187 and ionomycin-induced cell death.

The impairment of neutrophil-mediated (mainly phagocytosis) *P. aeruginosa* killing was noted earlier (72). This could contribute to the vulnerability of CF patients to infections, but effect of high concentrations of NaCl on *P. aeruginosa-*induced NETosis was not known. We found that hypertonic saline suppresses *Escherichia coli* LPS- and *P. aeruginosa*-mediated NETosis. This reinforces the biological relevance of our study. For instance, LPS and *P. aeruginosa* are relevant in the induction of NOX2-dependent NETosis in CF (69, 73, 74). Bacterial exposure is also inextricably implicated in sepsis (75). NETosis induced by *S. aureus* was not suppressed in hypertonic saline condition, suggesting the possibility that Gram-positive bacteria may use other pathways of inducing NETosis (19, 21). Therefore, hypertonic saline-mediated suppression of NETosis could be relevant for the treatment of different diseases involving Gram-negative bacteria.

In summary, this study elucidates a mechanism by which hypertonic saline suppresses NETosis. Hypertonic saline-mediated suppression of NETosis could be explained by biological fundamental principles. In hypertonic condition, water comes out from the cell via osmosis, causing dehydration of neutrophils that attenuates neutrophil function to suppress ROS and subsequent NETosis, an inflammatory cell death (1, 2, 5, 8). Specifically, hypertonic NaCl suppresses NOX2-dependent PMA-, LPS-, and *P. aeruginosa*-mediated NETosis. We have also established that hypertonic saline promotes apoptotic neutrophil death. This new knowledge opens new doors to devising new therapies for treating NETosis-associated diseases.

## Ethics Statement

The Research Ethics Board (REB) of the Hospital for Sick Children has approved the study protocols. All the healthy volunteers signed informed consent. All related methods were performed in accordance with the REB guidelines and regulations.

## Author Contributions

MK conceived the study. AN, JC, and MK conducted the experiments, prepared the figures, interpreted the data, and edited the manuscript. AN and MK wrote the manuscript. AF performed some experiments during the revision of the manuscript, and edited the manuscript.

## Conflict of Interest Statement

The authors declare that the research was conducted in the absence of any commercial or financial relationships that could be construed as a potential conflict of interest.
