# Supplementary material for: Hypertonic Saline Suppresses NADPH Oxidase-Dependent Neutrophil Extracellular Trap Formation and Promotes Apoptosis
Source: Front Immunol. 2018 Mar 8;9:359. doi: 10.3389/fimmu.2018.00359 (PMC5859219; doi:10.3389/fimmu.2018.00359)
Supplement: Supplementary file 1 [file image_1.PDF]

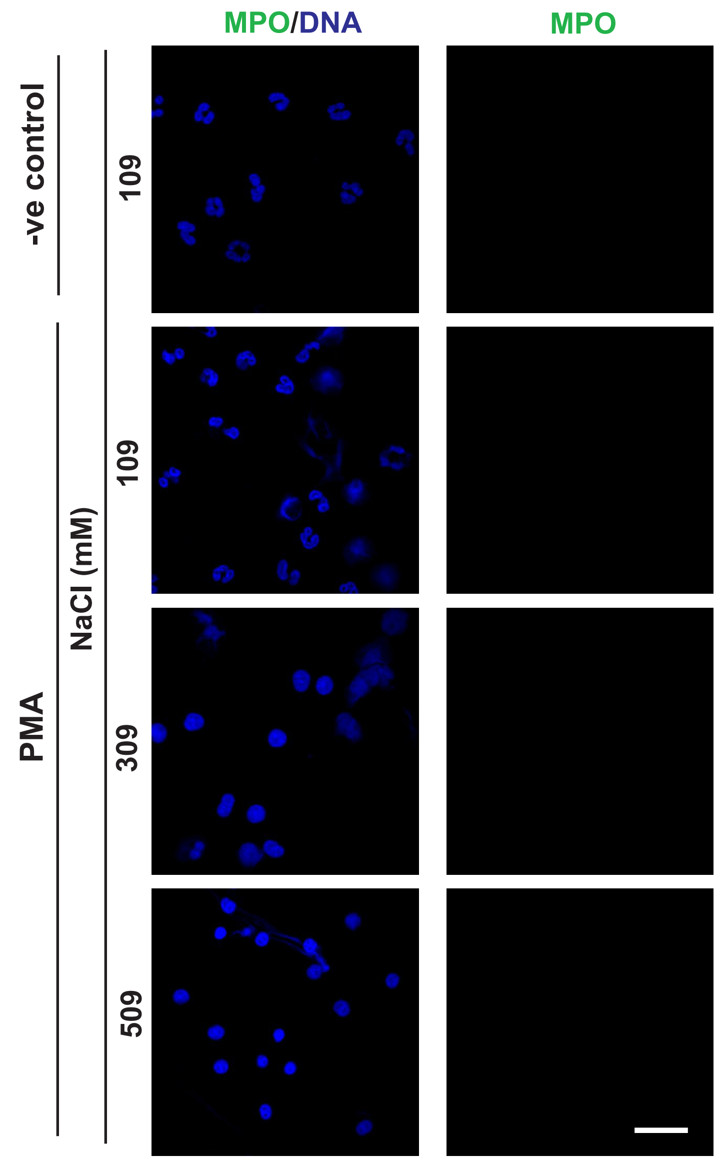

Figure S1. Isotype control for MPO immunostaining. Neutrophils were treated for the immunostaining as described in Figure 1 C. With minor modification, here cells were incubated with mouse IgG-Thermo Fisher®- (instead of MPO) isotype controls as primary antibodies. After 1h incubation followed by 2 washes with PBS 1X, neutrophils were incubated with secondary antibodies and DAPI. Blue=DAPI staining for DNA; Green=mouse IgG isotype control (to MPO); scale bar 22 μm.
