# Supplementary material for: Hypertonic Saline Suppresses NADPH Oxidase-Dependent Neutrophil Extracellular Trap Formation and Promotes Apoptosis
Source: Front Immunol. 2018 Mar 8;9:359. doi: 10.3389/fimmu.2018.00359 (PMC5859219; doi:10.3389/fimmu.2018.00359)
Supplement: Supplementary file 2 [file image_2.PDF]

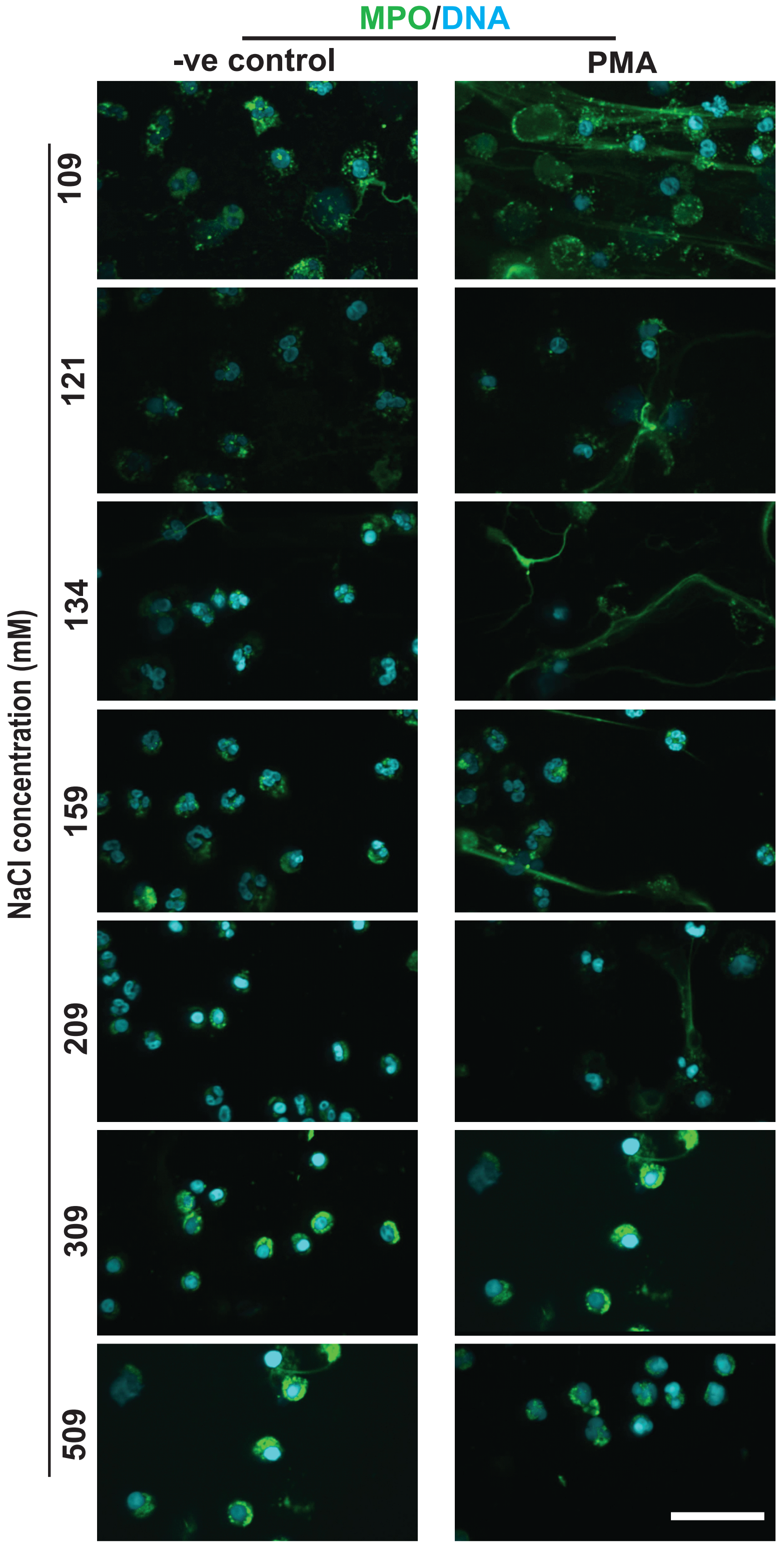

Figure S2. Confocal images show the suppressive effect of increasing NaCl concentrations during PMA-mediated NETosis. Immunostaining of the MPO already described in Figure 1C. The –ve control neutrophils show minimal MPO-NETs signals. Higher concentrations of NaCl treatments also show minimal MPO-DNA co-localization signals. Minimal MPO signal is detected for the 309 and 509 mM treatment, as NETosis is suppressed (n=3-4; MPO, green; DNA,DAPI-blue; scale bar, 22 μm).
