# Supplementary material for: Hypertonic Saline Suppresses NADPH Oxidase-Dependent Neutrophil Extracellular Trap Formation and Promotes Apoptosis
Source: Front Immunol. 2018 Mar 8;9:359. doi: 10.3389/fimmu.2018.00359 (PMC5859219; doi:10.3389/fimmu.2018.00359)
Supplement: Supplementary file 3 [file image_3.PDF]

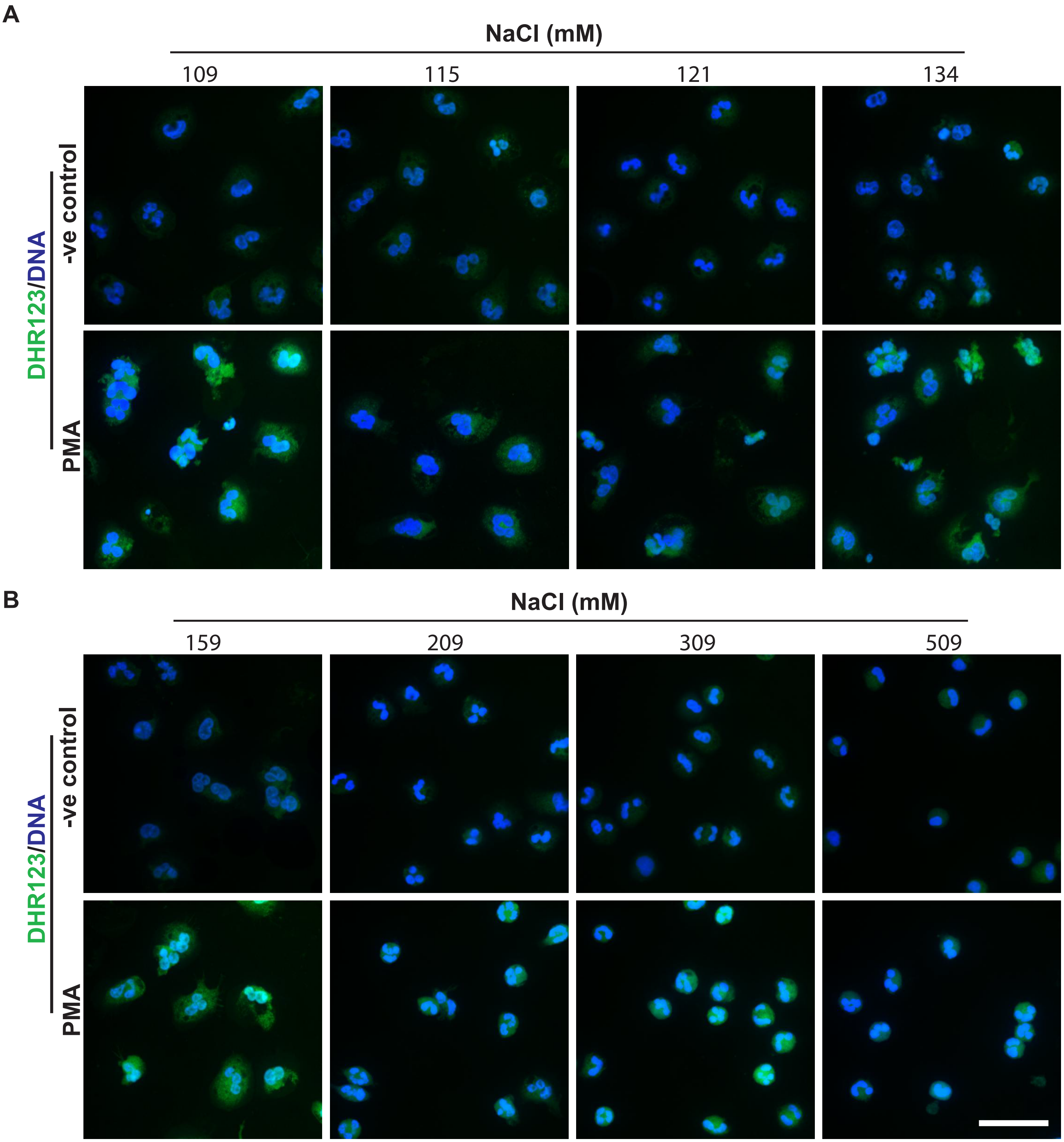

Figure S3. Increased NaCl concentration suppresses PMA-mediated ROS production. (A-B) Neutrophils were stained with DHR123 for ROS production estimation (fluorescence activity). After 1-hour incubation period, neutrophils were fixed and counter stained with DAPI. The -ve control neutrophils did not show obvious ROS staining. While, PMA treated neutrophils demonstrates a decrease in ROS production as NaCl concentrations increase compared to normal 109 mM saline condition. Minimal ROS production was seen for the 509 mM treatment (n=3-4; DHR123, green; DNA, DAPI; scale bar, 22 µm).
