# Supplementary material for: Hypertonic Saline Suppresses NADPH Oxidase-Dependent Neutrophil Extracellular Trap Formation and Promotes Apoptosis
Source: Front Immunol. 2018 Mar 8;9:359. doi: 10.3389/fimmu.2018.00359 (PMC5859219; doi:10.3389/fimmu.2018.00359)
Supplement: Supplementary file 4 [file image_4.PDF]

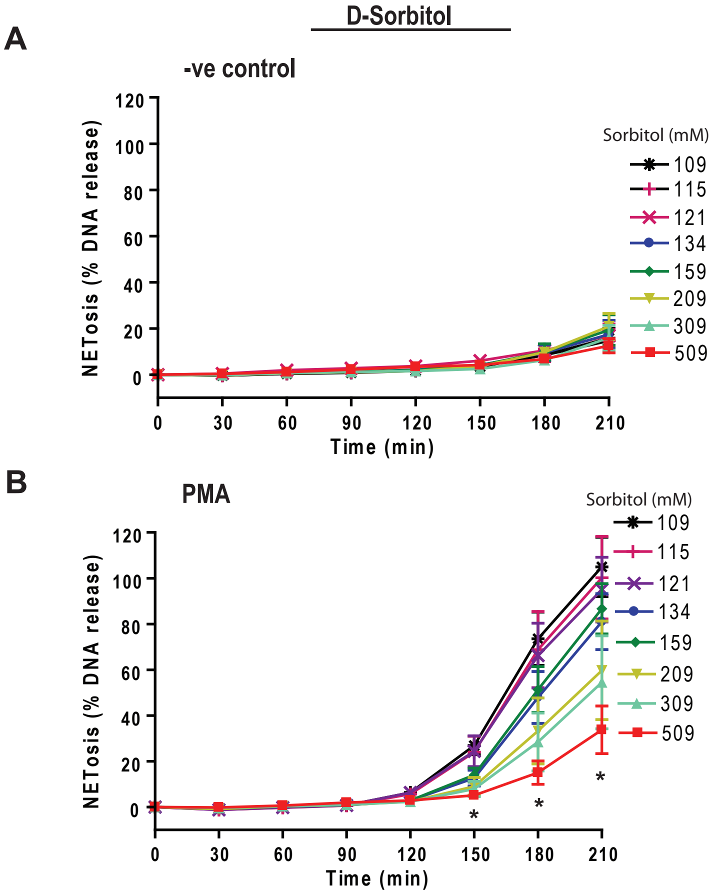

Figure S4. Increasing D-sorbitol concentration suppresses PMA-mediated NETosis. NETosis kinetics of neutrophils induced by either media only (-ve control) or PMA in different D-sorbitol concentrations was assessed. **(A-B)** Increasing concentration of the sorbitol suppresses PMA-mediated NETosis in dosage-dependent manner (n=3; *, $p<0.05$; Two-way ANOVA with Bonferroni's multiple comparison post test).
