# Supplementary material for: Hypertonic Saline Suppresses NADPH Oxidase-Dependent Neutrophil Extracellular Trap Formation and Promotes Apoptosis
Source: Front Immunol. 2018 Mar 8;9:359. doi: 10.3389/fimmu.2018.00359 (PMC5859219; doi:10.3389/fimmu.2018.00359)
Supplement: Supplementary file 5 [file image_5.PDF]

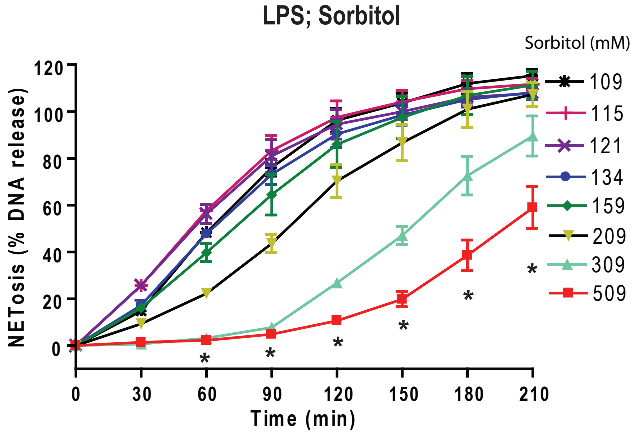


**Figure S5. Increasing D-sorbitol concentrations suppresses LPS-mediated NETosis.** NETosis kinetics of neutrophils induced by LPS in different D-sorbitol concentration were assessed. Increasing concentration of the sorbitol suppresses LPS-mediated NETosis in dosage-dependent manner (n=3; *, $p<0.05$; Two-way ANOVA with Bonferroni's multiple comparison post test).
