# Supplementary material for: Hypertonic Saline Suppresses NADPH Oxidase-Dependent Neutrophil Extracellular Trap Formation and Promotes Apoptosis
Source: Front Immunol. 2018 Mar 8;9:359. doi: 10.3389/fimmu.2018.00359 (PMC5859219; doi:10.3389/fimmu.2018.00359)
Supplement: Supplementary file 6 [file image_6.PDF]

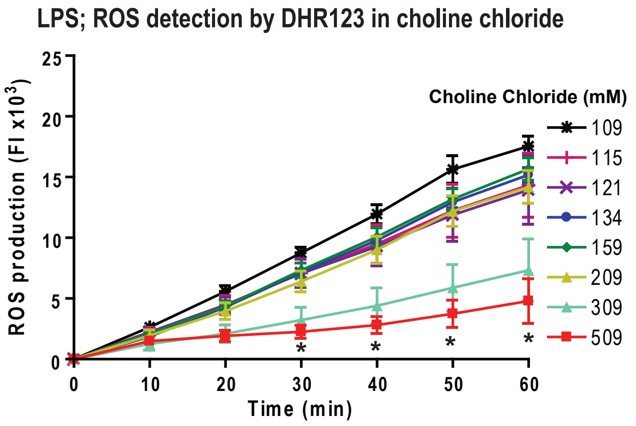

Figure S6. Increasing choline chloride concentrations suppresses LPS-mediated ROS production. The kinetics of the ROS production by neutrophils activated with LPS in different ChlCl concentrations was performed. ROS production during the treatment of 309 and 509 mM of ChlCl shows significant suppression (n=3; *, $p<0.05$; Two-way ANOVA with Bonferroni's multiple comparison post test).
