# Supplementary material for: Hypertonic Saline Suppresses NADPH Oxidase-Dependent Neutrophil Extracellular Trap Formation and Promotes Apoptosis
Source: Front Immunol. 2018 Mar 8;9:359. doi: 10.3389/fimmu.2018.00359 (PMC5859219; doi:10.3389/fimmu.2018.00359)
Supplement: Supplementary file 7 [file image_7.PDF]

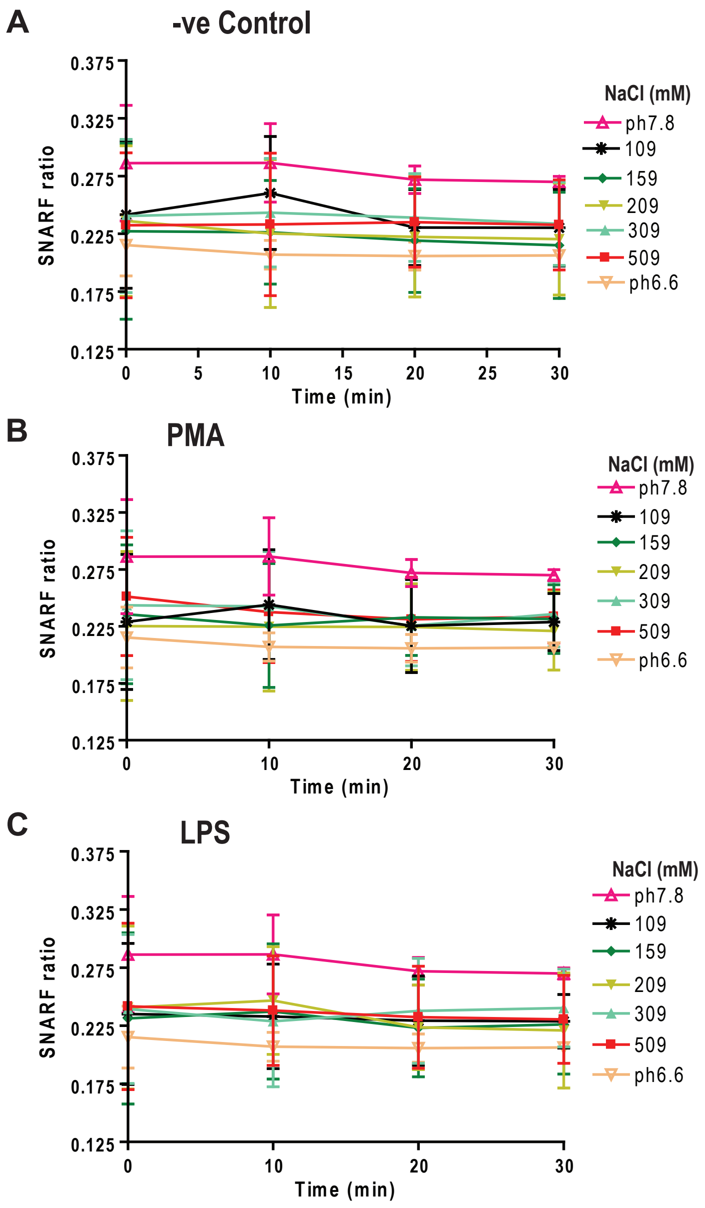

Figure S7. Hypertonic NaCl does not alter intracellular pH of neutrophils. pH of media was measured using the Carboxy SNARF-4F dye in a plate-reader assay. The emission spectra of the SNARF dye changes due to change in the pH and give two emission peaks. This dye typically used by exciting at one wave length (between 488 nm and 530 nm), while monitoring the fluorescence emission at two wave lengths (580 nm and 640 nm). The ratio of the fluorescence intensities of two emission wavelengths 640/580 nm were used as a proxy for intracellular pH measurement. **(A-C)** Different NaCl concentrations did not alter the pH of media in –ve control, PMA-treated cells or LPS-treated cells (n = 3; *, $p<0.05$; Two-way ANOVA with Bonferroni's multiple comparison post-test).
