# Supplementary material for: Hypertonic Saline Suppresses NADPH Oxidase-Dependent Neutrophil Extracellular Trap Formation and Promotes Apoptosis
Source: Front Immunol. 2018 Mar 8;9:359. doi: 10.3389/fimmu.2018.00359 (PMC5859219; doi:10.3389/fimmu.2018.00359)
Supplement: Supplementary file 8 [file image_8.PDF]

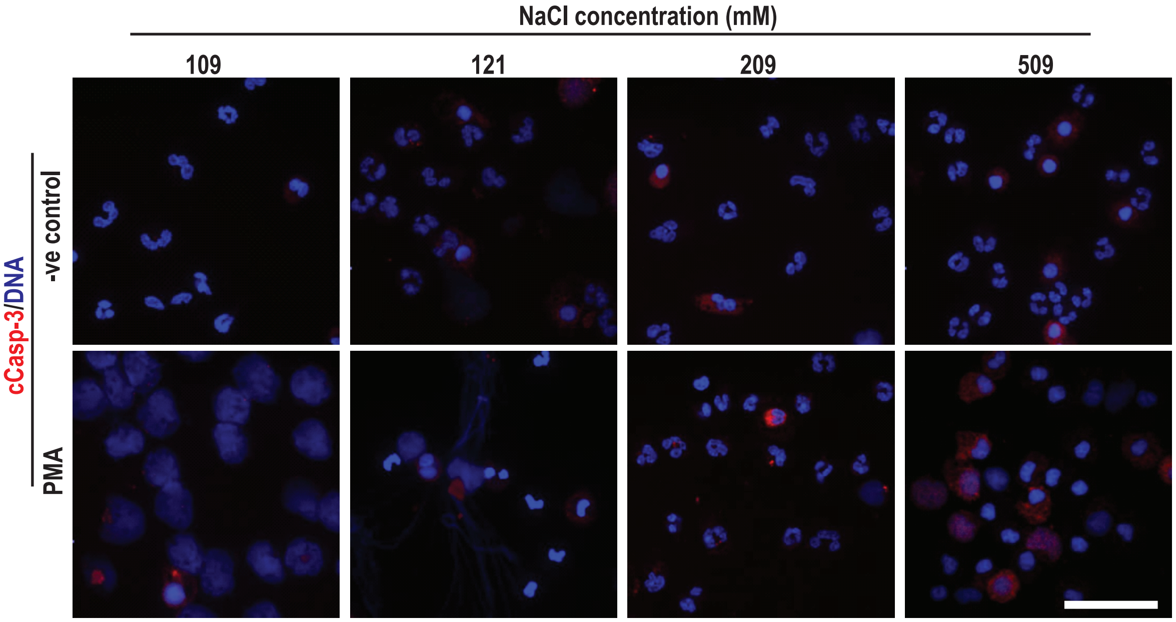

Figure S8. A high NaCl concentration switches PMA-mediated NETosis to apoptosis. Neutrophils were stained for cleaved Caspase 3 (cCasp-3), which represents apoptosis. Increased cCasp-3 correlated with increased NaCl concentration. These are the low magnification images shown in Figure 7 (n=3-5; cCasp-3, red; DNA, DAPI blue, scale bar, 22 µm).
